# Neodymium and Yttrium Adsorption on Citrate-Modified Cellulose: Experimental and Computational Insights

**DOI:** 10.1021/acsomega.5c07380

**Published:** 2026-01-23

**Authors:** Alessio C. Perri, Giorgio De Luca, Nasser AL-Hamdani, Vincenzo Algieri, Emilia Furia, Elpida Piperopoulos, Giuseppina Anna Corrente, Amerigo Beneduci

**Affiliations:** † Department of Chemistry and Chemical Technologies, 18950 University of Calabria, Rende, CS 87036,Italy; ‡ Institute on Membrane Technology, ITM-CNR, Ponte P. Bucci, cubo 17/c, Rende, CS 87036,Italy; § Engineering Department, 18980 University of Messina, C.da di Dio, 98166 Messina, Italy

## Abstract

Neodymium (Nd) and yttrium (Y), two rare earth elements, play a crucial role in a wide range of technologies, and their separation is a challenging process. Adsorption-based approaches offer a sustainable and cost-efficient substitute for the most widely used solvent extraction procedure. Here, we assess the potential of cellulose citrate (CC) as an adsorbent for the removal of Y and Nd through both experimental and computational approaches. CC was successfully synthesized by reacting raw cellulose extracted from *Spartium junceum* (Spanish broom) with molten citric acid using a green approach that does not require any solvent. The final goal is to shed light on the mechanism of adsorption by citrate-functionalized cellulose by interpreting the adsorption measurements through kinetics and isotherm adsorption models, as well as Density functional theory (DFT) calculations and molecular mechanics (MM) simulations. Adsorption properties of the sorbent are investigated at different contact times, pH values, and metal concentrations. Cellulose citrate has proven to be a highly effective material for the adsorption of the two metals, exhibiting a slight preference for Y at low-to-medium concentrations and for Nd at higher concentrations, suggesting a different binding stoichiometry of the two cations. The adsorption process is found to be pH-dependent, with equilibrium being reached after approximately 60 min. Interestingly, a certain degree of selectivity toward Nd is observed, which becomes more pronounced at pH values below 3 and at higher metal concentrations. DFT and MM modeling confirm the experimental results and allow an adsorption mechanism to interpret the measured performance of this material.

## Graphical abstract

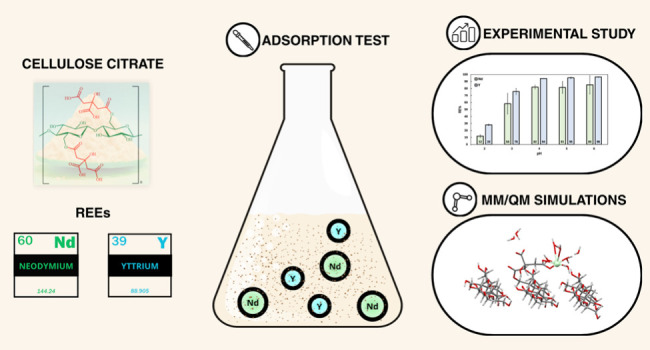

## 1 Introduction

Rare earth elements (REEs) find diverse applications in several fields, including catalysts, alloys, permanent magnets, optics, phosphors, sensors, and lasers.^1–3^ Specifically, neodymium (Nd) and yttrium (Y) hold significant importance in the global economy as they are used to produce powerful permanent magnets that are essential for the renewable energy industry.^4,5^

Separating rare earth elements (REEs), especially lanthanides, is a current challenge.^6^ Over time, advanced techniques have been developed, including solid-phase extraction, coprecipitation, membrane processes, ion-exchange, and chelation-induced adsorption. However, solvent extraction remains the most used method for separating REEs from their original mineral sources.^6–9^ While solvent extraction can be effective, it has notable limitations, especially when dealing with low metal aqueous concentrations. Moreover, this process often involves the use of hazardous compounds such as strong acids and organic solvents,^10^ leading to the generation of hazardous byproducts and waste materials.^11,12^ Conversely, adsorption-based approaches offer a sustainable, innocuous, and cost-efficient substitute for solvent extraction.^6^ These methods are particularly advantageous for solutions with low concentrations due to greater availability, scalability, and ease of implementation. Adsorption techniques have successfully removed REEs from various liquid solutions, including brines, saltwater, and metallurgical effluents.^13–19^

The selectivity of yttrium (Y) versus neodymium (Nd) in adsorption processes is crucial for the effectiveness of their separation. Unfortunately, their separation is very challenging since they have very similar chemical properties because of the identical electronic configuration of the outer electrons.^20^ Moreover, the coordination chemistry of rare earth metal ions is more limited compared with that of d-block transition metals. Stable complexes form mainly with strongly chelating ligands, especially those with highly electronegative donor atoms like oxygen.^21,22^ This limitation is due to the electronic configuration of rare earth ions: their 4f electrons are shielded and do not effectively participate in bonding, unlike the d-electrons in transition metals. As a result, bonding is primarily electrostatic, resembling that of alkaline earth metals rather than transition metals. The size of the cation also plays a significant role in determining complex stability and formation, with complex stability generally increasing with decreasing ionic radius for a given ligand.^21^ The distinct chemical behaviors of these elements can therefore be attributed to variations in their ionic radii, coordination chemistry, and electronic configurations.^21,23^ Yttrium, due to its smaller ionic radius, has a tendency to create complexes that are denser and more inflexible. On the other hand, Neodymium, with its greater ionic radius, favors forming complexes that are stronger and can accommodate larger and more adaptable ligands.^24,25^

Recent advancements in functional materials highlighted the importance of surface functionalization in enhancing the selectivity of adsorbents.^6,26–30^ Materials such as ion-imprinted polymers,^31^ porous organic frameworks,^32^ and silica-based adsorbents with covalently attached ligands^33^ are promising in selectively recovering REEs from complex mixtures. Furthermore, the use of green and hybrid adsorbents, incorporating sustainable and environmentally friendly components, is also gaining traction, offering high selectivity and efficiency with minimal environmental impact.^34–36^

However, the selectivity of Y and Nd is not just a matter of their chemical properties but also a function of the adsorbent materials. By leveraging the differences in their coordination chemistry and using advanced computational tools to guide the development of new materials, researchers can create highly selective and efficient adsorbents for a sustainable recovery of these critical rare earth elements.^37,38^

Several studies on the adsorption of Y and Nd on different materials have been published in the literature.^39^ Sorbents such as calcium alginate-poly glutamic acid hybrid gels (ALG-PGA),^40^ ion-imprinted mesoporous silica,^41^ magnetic nanohydroxyapatite adsorbent (MNHA),^42^ flower-like Nano-Mg­(OH)_2_,^43^ and EDTA- and DTPA-functionalized chitosan biopolymers,^27^ have demonstrated potential for Nd adsorption. Similarly, in the context of yttrium (Y) adsorption, different sorbents have shown promise, including spent bleaching clay impregnated with 3-amino-5-hydroxypyrazole (AHIBC),^44^ sodium alginate and calcium alginate compounds,^45^ titanium dioxide with surface arsenate groups (4As–TiO_2_), and titanium dioxide with surface arsenate groups doped by neodymium (Nd/4As–TiO_2_).^46^ Other studies have evaluated the adsorption of Y and Nd using the same adsorbent material. Among these, Guzzinati et al.^25^ investigated the recovery of the two metals on synthetic FAU zeolite X in its sodium form (NaX). They reported a maximum adsorption capacity (*Q*_*max*_) value of 9.52 × 10^–1^ and 1.32 mmol g^–1^ for yttrium and neodymium, respectively. The adsorption process was also evaluated on graphene oxide (GO)-based composite materials. Xu and co-workers synthesized these sorbents, such as HFG-GO (high gluten flour–graphene oxide)^47^ and 3D CEG.^48^ The latter was obtained by incorporating egg white into GO. The performance of the two composites is similar, with a comparable maximum adsorption capacity for both Y and Nd (0.36 and 0.34 mmol g^–1^). However, it is important to emphasize that almost all adsorption studies of rare earth elements have been performed by analyzing single-component solutions, i.e., solutions containing only one of the metals under investigation. Clearly, a comprehensive study of REEs separation must also include adsorption tests in multicomponent systems. To date, only a limited number of studies have focused on the selective adsorption of REEs, which represents a significant gap in the current literature. Selective adsorption experiments were conducted by Iftekar et al.^49^ who demonstrated that the GA5MA nanocomposite was especially selective toward scandium, while neodymium and yttrium exhibited a rather similar removal efficiency. In another work,^50^ the performance of various carbon-based materials in separating REEs was evaluated, achieving a Nd/Y mole fraction ratio of 1.6. These results further highlight the difficulty in separating these two metals.

In this study, we explored the potential of cellulose citrate (CC) as a selective adsorbent for Nd­(III) and Y­(III). CC was previously synthesized by reacting microcrystalline cellulose with molten citric acid.^51^ Here, we extend the synthesis using a raw cellulose biomass extracted from *Spartium junceum*, commonly known as *“Ginestra”*, which is a fast-growing vegetable,^52^ as a model for exploring the effective possibility of using any other cellulose source, even from waste. Owing to its biocompatibility and sustainability, CC is gaining attention as a promising material for metal adsorption. Functionalization with citric acid introduces multiple carboxyl groups, significantly enhancing the adsorption efficiency by increasing the number of accessible binding sites. A recent study^53^ demonstrated the material’s strong selectivity toward bivalent heavy metal cations, particularly mercury (Hg) and lead (Pb). Given its composition, renewable cellulose combined with the strong chelating properties of citric acid,^54^ CC presents a sustainable and effective approach for the separation of rare earth elements. To this end, we investigated the removal performance of CC in batch tests by determining the adsorption kinetics and isotherms for the two metal cations, both individually and in copresence, under varying conditions of the aqueous matrix. Additionally, advanced computational methods, such as density functional theory (DFT), were employed to elucidate the coordination environments and binding energies of the two REEs with cellulose citrate. This approach builds on previous studies,^55–57^ where DFT has proven effective in clarifying the adsorption mechanisms of REEs with various ligands. Azizi and Larachi^56^ employed DFT to simulate the efficiency of phosphonium-based bifunctional ionic liquids in extracting REEs. In a similar manner, Hu et al.^57^ utilized DFT to investigate the lanthanide selectivity when interacting with macrocyclic ligands. However, studies on the adsorption of Nd and Y on cellulose citrate are lacking. This study aims to address the above gap through detailed measurements characterizing the adsorption properties of CC, which are supported by first-principles and molecular mechanics (MM) calculations. The findings of the work will offer valuable references for designing efficient REE chelating sorbents and advancing the understanding of structure–properties relationships.

## 2 Materials and Methods

### 2.1 Materials

All chemicals were of analytical grade. All solutions were freshly prepared with bidistilled water and freed from any organic impurities by means of a Milli-Q system (Millipore, Burlington, MA, USA). The titrant carbonate-free solutions of sodium hydroxide (NaOH), perchloric acid (HClO_4_), and sodium perchlorate (NaClO_4_) were prepared and standardized as previously explained.^58^ For the potentiometric titrations, all of the test solutions were prepared by adding the appropriate amount of NaClO_4_, as a background electrolyte, to set the ionic strength at 0.7 M with the aim of avoiding variations during dilution.

Neodymium and yttrium solutions were prepared from the dissolution of Nd­(III) nitrate hexahydrate (Nd­(NO_3_)_3_·6H_2_O, Thermo Scientific, 99.9%) and Y­(III) nitrate hexahydrate (Y­(NO_3_)_3_·6H_2_O, Thermo Scientific, 99.9%) in HNO_3_ 1 wt %. The concentration of the two metals was determined by ICP-MS analysis (ICP-MS iCAP Q, Thermo Scientific) using the external standard method within the linear concentration range of 0.0069–3.45 μM.

Anhydrous citric acid (C_6_H_8_O_7_, H_3_L) was purchased by Merck. Cellulose citrate (R-H_2_L) was synthesized according to the procedure of Romeo and co-workers.^51^

### 2.2 Extraction of Cellulose from *Spartium junceum* and Synthesis of Cellulose Citrate

#### 2.2.1 Acid Pretreatment

Initially, the broom fiber is mechanically pulverized using a paddle mill. Subsequently, in a 3 L two-necked round-bottomed flask equipped with a bubble condenser, lignocellulosic material (200 g) is introduced and dispersed in 0.1 M HCl solution (1.5 L). The suspension is refluxed for 2 h under gentle stirring. After cooling to room temperature, the resulting solid is filtered, washed with distilled water to neutral pH, washed with acetone (100 mL), and dried overnight in an oven at 80 °C.

#### 2.2.2 Bleaching Material Process

In a 2 L two-necked round-bottomed flask, equipped with a mechanical stirrer, solid pretreated material is poured and dispersed in 1 L of aqueous solution consisting of 6% w/v H_2_O_2_ and 2% w/v NaOH. The suspension is stirred vigorously for 12 h at room temperature. The resulting solid is filtered, washed with water to neutral pH, washed with acetone (100 mL), and dried overnight in an oven at 80 °C. Yield: 65%.

#### 2.2.3 Synthesis of Cellulose Citrate (CC)

In a Pyrex glass beaker, 20 g of cellulose derived from *Ginestra* and 20 g of anhydrous citric acid are placed and mixed together. The mixture is heated at 152–155 °C, using an oil bath, for 3 h and mixed mechanically. At the end of the reaction, after cooling the reaction mixture to room temperature, 30 mL of acetone is added, and the suspension is filtered using a 125 mL sintered glass filter connected to an Erlenmeyer flask. The filtered solid was washed with distilled water (50 mL) and with 30 mL of acetone, dried in an oven at 70 °C for 1 day, and consisted of cellulose citrate as a pale-yellow solid. Yield: 45%.

### 2.3 Characterization of Cellulose Citrate

Cellulose citrate was characterized using a combination of spectroscopic, structural, surface area, and morphological analyses.

The functionalization reaction was verified by Fourier transform infrared (FTIR) spectroscopy using a Bruker ALPHA FTIR spectrometer equipped with an A241/D reflection module. FTIR spectra were acquired on dry disk pellets of the materials in the spectral range 375–4000 cm^–1^ with 48 scans per single run. The OPUS Bruker software was used for the interpretation of the recorded spectra.

Structural features and crystallinity changes induced by the modification were investigated through XRD analysis, performed using a Bruker D8 Diffractometer (Billerica, Massachusetts, USA), with copper Kα radiation (λ = 1.5418 Å) under 40 kV and 40 mA operating conditions. Scanning ranged from 2ϑ = 5° to 2θ = 50°, with a step speed of 0.037 °/s.

Textural properties were characterized by N_2_ adsorption–desorption at 77 K using a Quantachrome NOVA II instrument (v.11.02). Samples were degassed at 70 °C for 14 h before analysis. The specific surface area was calculated by the Brunauer–Emmett–Teller (BET) method, and pore volume and pore size distribution were derived from the Barrett–Joyner–Halenda (BJH) method, using the cross-sectional area of nitrogen (16.2 Å^2^).

Finally, the morphology and elemental composition of cellulose citrate before and after adsorption, Nd-CC and Y-CC, were investigated by SEM-EDX with an FEI Quanta FEG 450 microscope. Samples were assembled on aluminum stumps using double-sided tape. An accelerated voltage of 10 kV was used for all samples under low vacuum conditions.

### 2.4 Potentiometric Titrations

Potentiometric measurements and titrations were carried out using the same apparatus described previously.^58^ Acid–base equilibria were investigated through potentiometric titrations conducted in a thermostated bath at (25.0 ± 0.1) °C, in a medium of 0.7 M NaClO_4_. The electromotive force (EMF) of cell (G) was measured using a Metrohm alkaline glass electrode (GE), configured as follows:

(G):RE|TestSolution|GE

where RE is the reference electrode (Ag|AgCl|0.01 M NaCl|0.7 M NaClO_4_). After reagent addition, a stable potential (within ± 0.01 mV) was reached within 25 min. The test solution contained C_L_ M H_3_L (or R-H_2_L), C_A_ M HClO_4_, C_B_ M NaOH, and 0.7 M NaClO_4_. Nitrogen commercial cylinder gas was moderately introduced into the magnetically stirred test solutions to prevent any interference due to carbonate formation. The gas was purified by passing it through 1 M NaOH, 1 M H_2_SO_4_, double-distilled water, and 0.7 M NaClO_4_. The experimental primary data (i.e., *C*_L_, *C*_A_, *C*_B_, [H^+^], *E*, and added volumes of titrant) were employed to obtain acidic constants. The protonation constants of citric acid and cellulose H_2_Citrate were determined with the computer program Hyperquad.^59^ Standard deviations are indicated as 3σ values (i.e., data within three standard deviations from the mean, covering 99.7% of the data). The EMF of the cell (G) can be expressed, in mV, according to eq 1:

$$E=E^{{}^{\circ}}+61.54\log\left[H^{+}\right]+E_{j}$$ 1

where *E°* was constant for each series of measurements and *E*_*j*_ is the liquid junction potential, which, under the selected experimental conditions, is a function of [H^+^] only.

At the beginning of all titrations, the *E*° constant value (within 0.05 mV) was determined in the [H^+^] range 10^–4^–10^–2^ M in the absence of ligands and in accordance with Gran’s method.^60,61^ In the first step, the acidity of the solution was gradually decreased by reducing water electrochemically, generating OH^–^ ions using a coulometric setup (C).

(C): Pt | Test Solution | AE^+^

where AE denotes the auxiliary electrode, composed of 0.7 M NaClO_4_/0.1 M NaCl, 0.6 M NaClO_4_/Hg_2_Cl_2_/Hg.

In the second step, following the addition of ligands, the acidity was further decreased by the stepwise addition of known volumes of a standard NaOH solution. The ligand concentration C_L_ was varied between 0.5 and 5 × 10^–3^ M, while the hydrogen ion concentration ranged from 10^–2^ to 10^–6^ M. All titrations were performed using a programmable, computer-controlled data acquisition switch unit (Model 34970A, Hewlett–Packard). The constant-current source for the coulometric circuit was provided by a DC power supply (Model 6614C, Hewlett-Packard). EMF values were measured with a precision of 10^–5^ V by using an OPA111 low-noise, precision DIFET operational amplifier.

### 2.5 Determination of the Number of Binding Sites

The number of binding sites was determined by a potentiometric acid–base titration. Specifically, 0.2 g of CC was suspended in 50 mL of ultrapure water. This was then acidified to pH 2 with 0.02 M HCl and backtitrated to pH 10 with 0.1 M NaOH. The end titration point was determined by using the first derivative method.

### 2.6 Adsorption and Reusability Experiments

All adsorption experiments were carried out at room temperature with 10 mg of cellulose citrate dispersed in 50 mL of solution of the metal of interest in test tubes that were agitated (350 rpm) using an ARGO LAB SKO–D XL agitator. The performance of the material was evaluated at different pHs, contact times, and initial metal concentrations. The effect of pH was investigated in a range of 2–6, while for the kinetic experiments, the adsorption properties of CC were monitored over time. Finally, solutions with concentrations in the range of 6.9–345 μM were prepared for the study of adsorption isotherms. Unless otherwise specified, experiments were carried out in triplicate at a metal concentration of 6.9 μM (1 mg L^–1^ Nd, 0.61 mg L^–1^ Y) and pH = 4.5, with samples analyzed after equilibrium was reached.

The adsorption process was evaluated in terms of removal efficiency (RE, %) and adsorption capacity at time *t* (*Q*_*t*_, μmol g^–1^), defined as

$$RE=\frac{C_{0}-C_{t}}{C_{o}}$$ 2

$$Q_{t}=\frac{\left(C_{0}-C_{t}\right)\times V}{m}$$ 3

where *C*_0_ is the initial metal concentration (μM), *C*_*t*_ is the metal concentration at time *t* (μM), *V* is the volume of solution (L), and *m* is the mass of adsorbent (g).

The reusability of the material was evaluated by adsorbing 50 mL of a 6.9 μM metal solution (pH = 4.5) onto 10 mg of cellulose citrate. The material was then regenerated using 10 mL of 0.1 N HCl for three different desorption times (10, 30, and 60 min). After each step, the material was filtered, thoroughly washed with ultrapure water, and finally dried. The procedure was repeated for two consecutive reuse cycles.

### 2.7 Selectivity

The selectivity of cellulose citrate toward Nd was tested by repeating the same experiments with both metals in solution, allowing them to compete for the same active sites. Thus, 10 mg of material was dispersed in a solution containing 6.9 M of both neodymium and yttrium. The selectivity was evaluated as a function of pH (at equilibrium) and contact time (pH = 4.5). Finally, experiments with three different metal concentrations, namely, 6.9, 34.5, and 69 μM, were carried out at pH = 4.5, analyzing the solution after reaching equilibrium. All of the tests were performed in duplicate.

The selectivity coefficient (*S* ^0^_Nd/Y_) was determined as the ratio of the distribution coefficient (*K_d_*) of Nd and Y, defined as

$$S_{\mathrm{Nd}/Y}^{0}=\frac{K_{d(\mathrm{Nd})}}{K_{d(Y)}};K_{d}=\frac{Q_{e}}{C_{e}}$$ 4

where *K*_*d*_ (L g^–1^) is the distribution coefficient of the considered metal between the CC and the solution; *Q*_*e*_ (μmol g^–1^) and *C*_*e*_ (μM) are the adsorption capacity and metal concentration at equilibrium.

### 2.8 Models and Computational Details

The cellulose citrate models were obtained through a combined approach based on molecular mechanics and density functional theory-based calculations. First, we prepared a single cellulose chain consisting of five glucose units, in both chair and boat conformations, then a systematic conformational search was carried out using the Universal Force Field (UFF) and VEGA ZZ 3.2.2 code.^62^ This preliminary study allowed a satisfactory screening of the large number of possible conformers and identification of the most stable structures among them. Each conformer of the cellulose chain was optimized using the conjugate gradient algorithm, with an energy convergence threshold of 10^–7^ and a maximum of 1000 geometries explored (steps) to locate the minimum-energy structure. The cellulose chain with the glucose in the chair conformation was found to be more stable with respect to the one with units in the boat conformation. A cellulose model was subsequently designed by assembling three chains in parallel, and this structure was then optimized at the quantum level (Table S1, Figure S1A). The citrate functional group was designed by performing a systematic conformational search on the citric acid methyl ester using the previous MM protocol. The most stable conformer coming out from the conformational search (Table S2, Figure S1B) was optimized at the DFT level (Table S3, Figure S1C). Finally, the optimized citrate was attached to the glucose CH_2_OH located at the center of the fiber in order to obtain a functionalized cellulose model, which was again optimized at the quantum level.

#### 2.8.1 DFT Calculations

The quantum calculations were performed using the Northwest Computational Chemistry Package (NWChem)^63^ and the B3LYP exchange-correlation functional.^64^ Coulomb and exchange-correlation potentials were numerically integrated on an adaptive grid with accuracy equal to 10^–5^, while the threshold for the energy convergence and the root-mean-square of the electron density were set to 10^–6^ (a.u.) and 10^–5^ (a.u.), respectively. No smear and level shift values were imposed. Closed-shell configurations for citrate, cellulose, and the CC model, and all Yttrium complexes were used, while a quadruplet electronic multiplicity for the Nd complexes was used according to previous results.^65^ Dispersion contribution to the total DFT energy was calculated according to Grimme’s DFT-D4 approach.^66,67^

Different Gaussian-type orbitals were used. In detail, the 6–31G* basis set was employed for the citric acid methyl ester and the cellulose model. As for metal complexes, the 4–31G basis set on the C, H, and O atoms of the cellulosic support and 6–31G* on the citrate functional group were used in addition to the Stuttgart RSC 1997 ECP for Nd and Y. Considering the number of systems analyzed, a trade-off between computational time and results accuracy was necessary. For this reason, an MM/QM approach has been adopted for the cellulose citrate-metal complex geometry optimizations. In detail, the atoms of the cellulose support were replaced with point charges, while the functional group chelating the metals was treated at the DFT level. The point charges were calculated on the full DFT-optimized geometry of CC, through the Electrostatic Potential approach (EPC) implemented in the NWChem code, in which the atomic charges of the system are optimized to reproduce the quantum potential on a fine grid. The NWChem Driver module was used for geometry optimization, adopting the default thresholds for maximum and root-mean-square gradients as well as for maximum and root means atoms displacements.

The adsorption energies, Δ*E*_*ads*_, of the Nd and Y on the functionalized cellulose at the solid/solution interface were calculated from the well-known relation:^68^

$$\begin{array}{c} \Delta E_{ads}=E_{CCM}-E_{M}-E_{CC}\\ \mathrm{with}\;E_{M}=E_{M}(\mathrm{vacuum})-\Delta E_{M}(\mathrm{hyd}) \end{array}$$ 5

where *E*_*CCM*_ is the DFT energy referred to the cellulose citrate-metal complex, while *E*_*M*_(vacuum) and *E*_*CC*_ are the energies of metal and cellulose citrate noninteracting. *E*_*CCM*_, *E* *M*, and *E*_*CC*_ were calculated using the optimized geometries of the cellulose citrate-metal complexes at the DFT level in single-point calculations through a high level of theory considering 6–311G* basis set. Δ*E*_ads_ is calculated assuming noninteracting fragments that constitute the target CC-M complex. The metal hydration, i.e., the coordination of the metal with H_2_O molecules, has been included in the calculation directly through the term Δ*E*_*M*_(hyd), which is the difference between the energy of the hydrated metal complex and the energies of noninteracting metal and water molecules (eq 5). In other words, it takes into account the energy released by the hydrated metal complex with the water molecules of the first coordination shell. Six water molecules were considered in the hydrated complex as obtained from previous calculations.^65^ Although in the literature, Δ*E_ads_* is often reported without considering the Δ*E*_*M*_(hyd) contribution,^26,69,70^ it has been observed that including this correction leads to better agreement with the experimental results.^68^

## 3 Results and Discussion

### 3.1 Synthesis

*Spartium junceum* (*Ginestra*) is a perennial shrub native to the Mediterranean region. It has long been valued not only for its ornamental and ecological properties but also for its strong and flexible fibers.^71^ In recent years, interest has grown in using *Ginestra* as a sustainable source of cellulose, a key component for producing paper, textiles, bioplastics, and other biobased materials. The extraction of cellulose from *Spartium junceum* involves several stages (Scheme 1A).

**Scheme 1:**
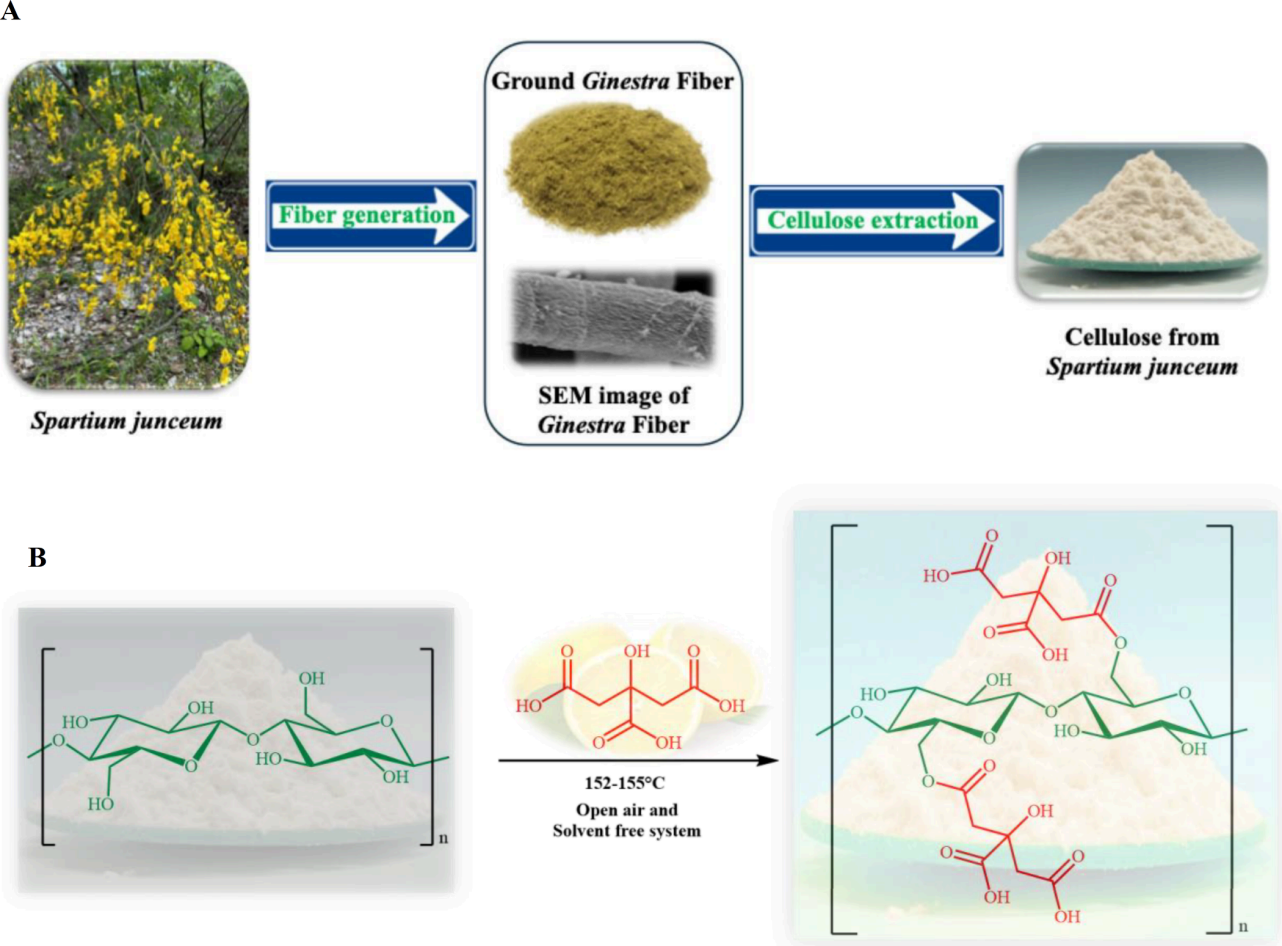
(A) Scheme of the Cellulose Extraction Process from *Spartium junceum* (*Ginestra*) and (B) of the Synthesis of Cellulose Citrate (CC) with Citric Acid

First, the plant stems are harvested and subjected to a retting process, either by water or by chemical means, to separate the fibrous material from the woody core. Once the fibers are isolated, they undergo a purification process to remove noncellulosic components such as lignin, hemicellulose, and waxes.^52^ This is typically achieved through a combination of alkaline treatments (e.g., with sodium hydroxide) and bleaching steps using hydrogen peroxide or similar agents.^72^ The result is a purified cellulose pulp with good mechanical properties, suitable for various industrial applications. Compared to traditional sources, such as wood or cotton, *Ginestra* offers several advantages: it grows well in poor soils, requires little water, and does not compete with food crops.

The simultaneous transformation of cellulose into cellulose citrate was realized with our developed sustainable procedure,^51^ avoiding the use of strong inorganic acids, drastic conditions, enzymatic treatments, or microorganism fermentation. This innovative method is very eco-friendly and involves the use of molten citric acid under solvent-free conditions at atmospheric pressure according to Scheme 1B.

Cellulose and pure citric acid were mixed in a 1:1 weight ratio and heated in an open reaction system at 152–155 °C using an oil bath for 3 h, with occasional mechanical stirring. The resulting solid was sequentially washed with water and then acetone, and finally dried.

### 3.2 Chemical Physical Characterization of the Adsorbent

The FTIR spectrum (Figure 1A) of raw cellulose displays characteristic bands at: 600–900 cm^–1^ for C–O–H, C–O–C, C–C–O, and C–C–H deformation modes and C5/C6 stretching vibrations; 1100–1400 cm^–1^ for C–H deformation; 1430 cm^–1^ for H–C–H and O–C–H in-plane bending; 2900 cm^–1^ for C–H stretching; and 2995–4000 cm^–1^ for hydrogen-bonded O–H stretching. The FTIR spectrum of CC retains all major features of pristine cellulose, with an additional strong absorption band at 1736 cm^–1^, attributed to the carboxyl group stretching of citric acid, confirming the successful functionalization (Figure 1A).^51^

**Figure 1:**
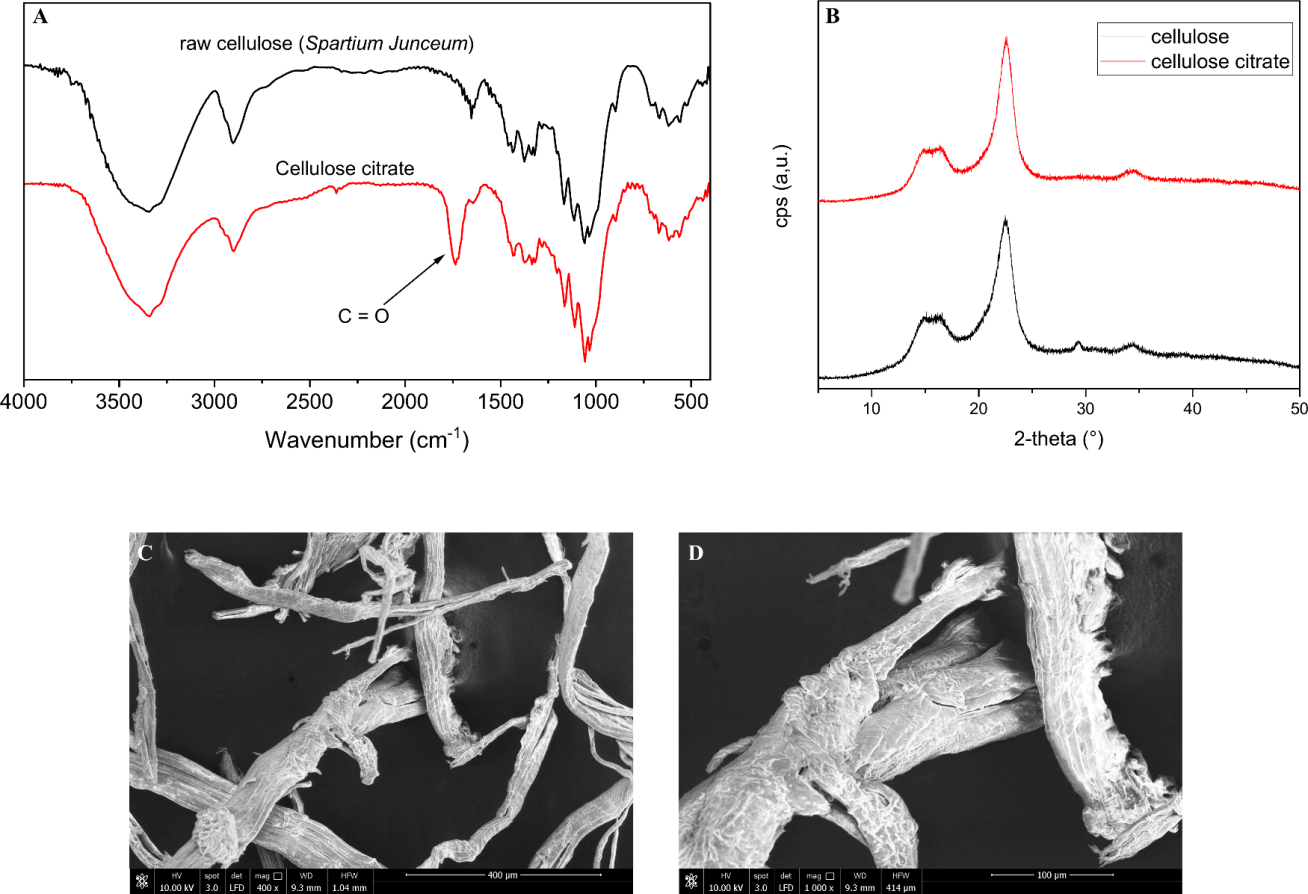
(A) FTIR spectra of raw cellulose extracted from *Spartium junceum* and cellulose citrate. (B) XRD diffractograms of cellulose (black curve) and cellulose citrate (red curve). SEM images of cellulose citrate at different magnification (C, D).

Figure 1B shows the diffractograms of cellulose and modified cellulose. All samples resulted in similar patterns, with peaks compatible with the characteristic pattern of cellulose at 2ϑ = 15, 22, and 34°.^52,73,74^ The process did not change the crystalline organization of cellulose.

This finding is further supported by the SEM morphological analysis, evidencing that cellulose citrate (Figure 1C,D) retains all the characteristic features of pristine cellulose, showing long and individualized fibers.^52,73^

Figure 2A,B shows nitrogen adsorption–desorption isotherms for pristine cellulose and cellulose citrate. The BET analysis of cellulose shows a relatively low specific surface area of 6.97 m^2^/g, with a pore half-width centered at 14.48 Å and mainly ranging from 10 to 40 Å (Figure 2C). The cumulative pore volume is 0.09 cm^3^/g (Figure 2C). These values indicate that cellulose exhibits predominantly mesoporous characteristics, with medium-to-small pores and a relatively open structure, typical of amorphous cellulose with intrinsic hydrogen bonding between chains.^75,76^ After functionalization with citrate groups, a slight increase in the specific surface area is observed, reaching 9.95 m^2^/g, along with a more concentrated and uniform pore distribution centered at 16 Å and mostly ranging from 14 to 20 Å (Figure 2D). The cumulative pore volume slightly decreases to 0.07 cm^3^/g (Figure 2D). This behavior suggests that citrate functionalization leads to a slight increase in surface area and introduces additional chemically active sites capable of interacting with polar molecules or ions despite a slight decrease in the total pore volume. The narrower pore distribution indicates a “structuring” effect on the cellulose surface, likely due to the incorporation of citrate groups, which limit the formation of larger pores and increase the uniformity of the microstructure.^3,4,77,78^

**Figure 2:**
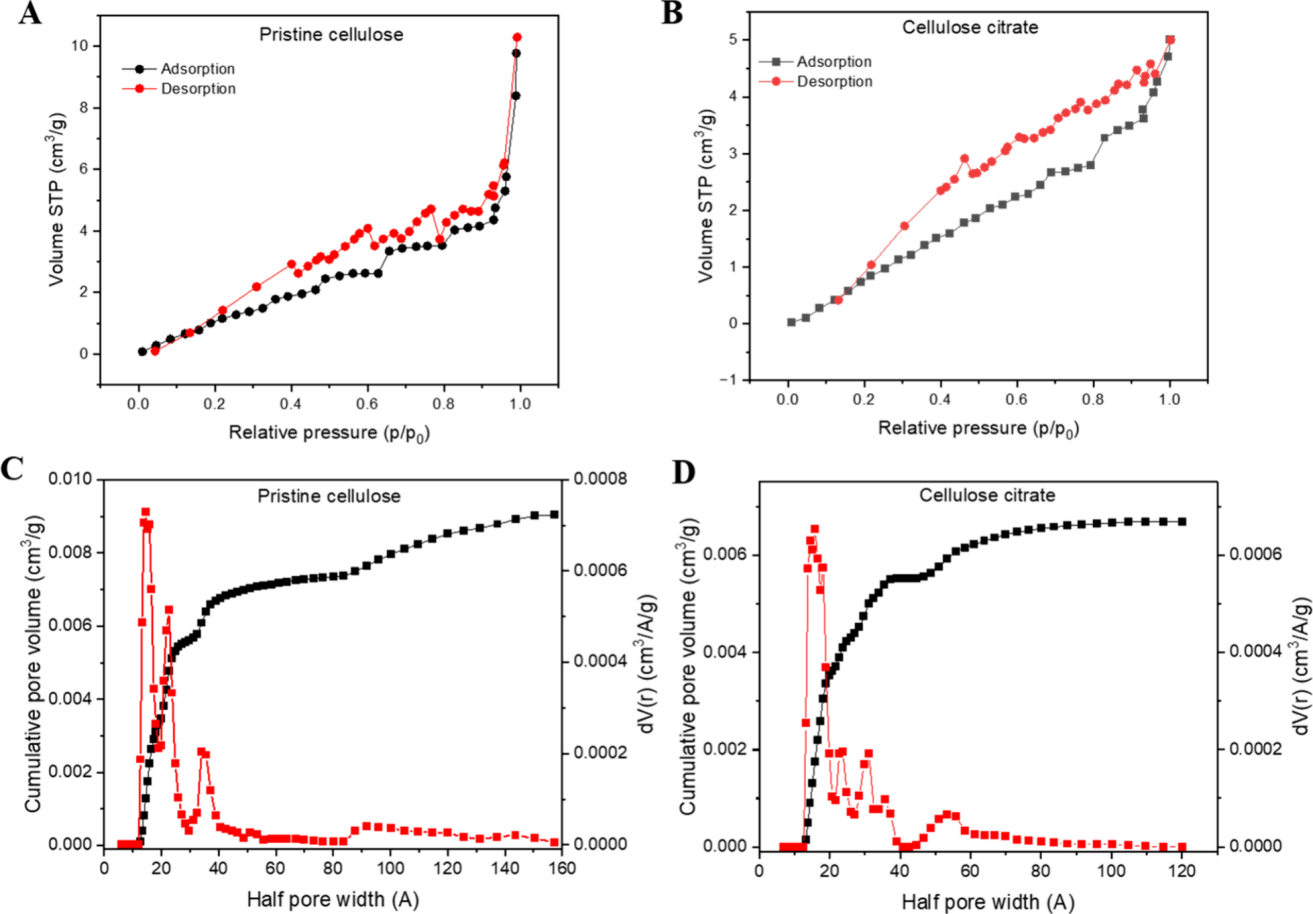
(A) N_2_ adsorption–desorption isotherms (77 K) of pristine cellulose and (B) cellulose citrate. Pore size distribution graph of (C) pristine cellulose and (D) cellulose citrate.

### 3.3 Acidic Constants of Citric Acid and Cellulose Citrate

Accurate results were acquired by taking into consideration the physical integrity of ligands and their stability at different pH values during the whole experiment. Therefore, the studied systems were kept under an inert atmosphere and shielded from light radiation. The potentiometric data, recorded at 25.0 °C and in 0.7 M NaClO_4_ and obtained by three titrations for each compound, were treated by numerical as well as by graphical methods^59,79^ and were fully explained by the general equilibrium reported in reaction 1:

$$H_{n}L+H_{2}O⇄H_{n-m}L^{-m}+mH^{+}\log\;\beta_{m}(\pm 3\sigma )$$ R1

To facilitate a comparison with literature data, results obtained by numerical treatment are reported in Table 1 as stepwise p*K*_a_ *_m_* values.

**Table 1:** *pK*_*m*_ Values, According to the General Equilibrium Reported in Reaction 1 for Citric Acid (H3L) and Cellulose Citrate (R-H_2_L) Obtained at I = 0.7 M NaClO_4_ and 25 °C (Standard Deviations Represent 3σ)

H_3_L	p*K*_a1_	p*K*_a2_	p*K*_a3_
	1.9 ± 0.1	3.7 ± 0.1	5.1 ± 0.1
R-H_2_L	p*K*_a1_ + p*K*_a3_		
	6.6 ± 0.1		

With regards to cellulose citrate, we obtained a value which, within the experimental error, is equal to the sum of the p*K*_a1_ and p*K*_a3_ of citric acid; therefore, it can be assumed that the esterification of citric acid with cellulose occurs on one of the −COOH groups, which is less sterically hindered.

The trend of experimental points was also evaluated by a graphical approach representing *Z*_H_ *,* obtained at different ligand concentration (*C*_L_) values, with respect to pH. The function *Z*_H_ corresponds to the mean number of protons released for ligand,^79^ and it is equal to ([H^+^] – *C*_A_ + *C*_B_ + *K*_w_/[H^+^])/*C*_L_, in which *C*_A_ and *C*_B_ are the analytical concentrations of HCl and NaOH, respectively. For the ion product of water, under our experimental conditions, we have used values from the literature.^80^ In Figure S2A,B, the graphical evaluation for H_3_L and R-H_2_L is reported, respectively.

For different *C*_L_ values, the experimental points overlap. By performing back-titration for cellulose citrate, we verified that the general equilibrium in reaction 1 explains the experimental data. A qualitative comparison with the literature p*K*_a_ values is possible only for citric acid (Table 2). The agreement of our data is barely satisfactory, especially for the p*K*_a*1*_ values.

**Table 2:** Experimental p*K*_a_ Values of Citric Acid from the Literature

	p*K*_a1_	p*K*_a2_	p*K*_a3_	references
H_3_L	1.9	3.7	5.1	this work
	2.95	4.13	5.54	^81^
	3.13	4.76	6.40	^82^

### 3.4 Adsorption Studies

#### 3.4.1 Effect of pH

Due to the presence of carboxyl groups in the structure of cellulose citrate, the pH of the metal solution becomes one of the main parameters influencing the adsorption process. In this study, the variation of removal efficiency (RE%) and adsorption capacity (*Q*_*e*_) at equilibrium in the pH range between 2 and 6 was evaluated. The results are listed in Figure 3.

**Figure 3:**
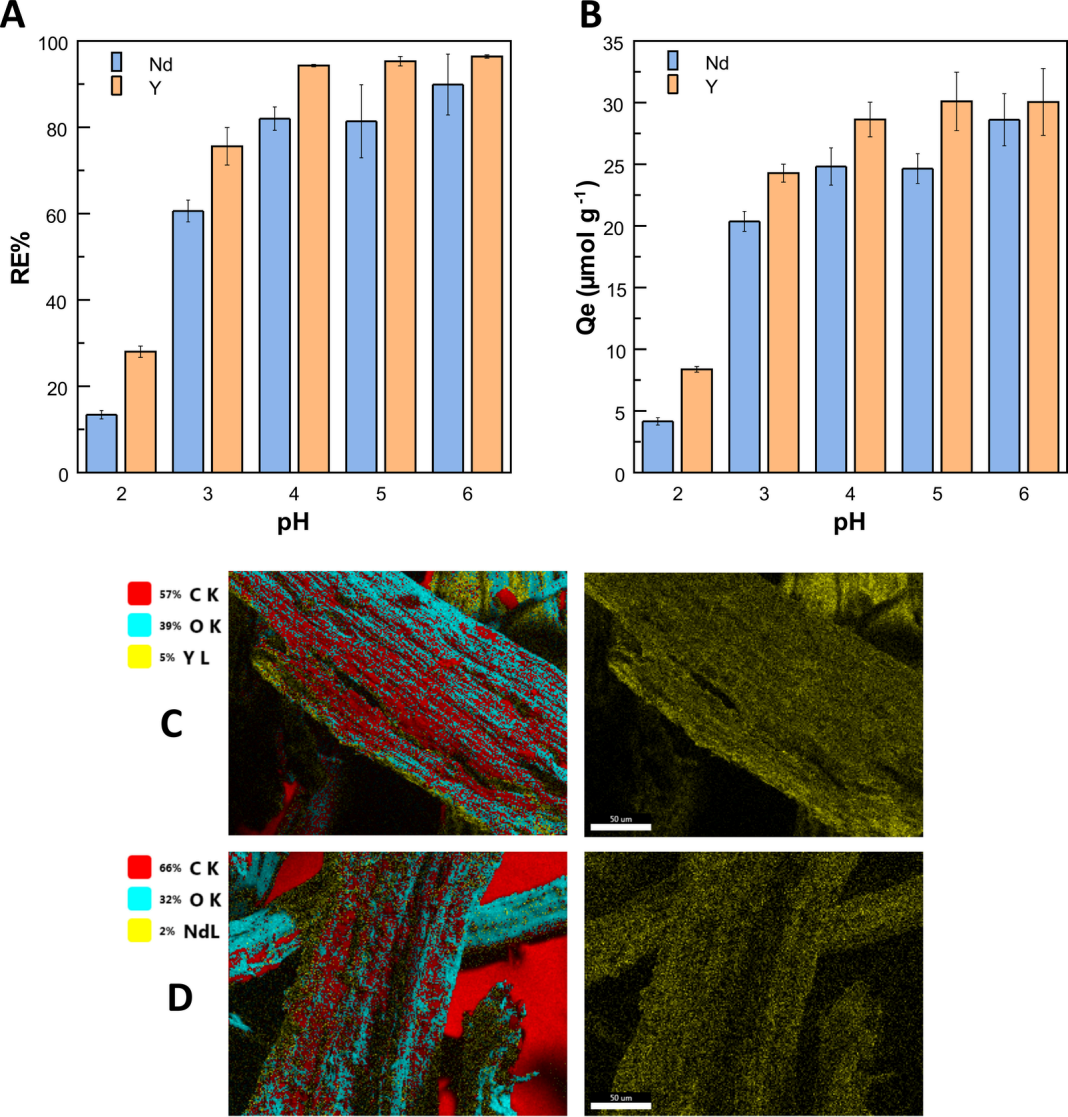
Effect of pH metal solution on the (A) removal efficiency (RE%) and (B) adsorption capacity (*Q_e_*). (C, D) Elemental mapping by SEM-EDX of cellulose citrate post adsorption of Y and Nd, respectively.

Overall, CC proves to be an efficient material for the adsorption of both metals, with yttrium being slightly more favored than neodymium. Removal efficiency and adsorption capacity increase with increasing pH. At pH 6, almost complete adsorption of the metals occurs, with RE values of (96.4 ± 0.4) and (90 ± 7) % for Y and Nd, respectively, and relative *Q*_*e*_ of (30 ± 3) μmol g^–1^ and (29 ± 2) μmol g^–1^. These results can be interpreted by considering the acid–base equilibria involving the carboxyl groups of cellulose citrate. As the pH value increases, the fraction of deprotonated acid sites increases, causing the surface of the material to become increasingly negatively charged. These conditions lead to a better interaction between the citrate functional group and metal cations, favoring their adsorption. The same behavior has been found in other studies.^18^ It is interesting to note that the enhancement in adsorption efficiency occurs in steps: a first jump is recorded between pH 2 and 3, while a second one occurs from pH 3 to 4. These data give an indication of the values of p*K*_a1_ and p*K*_a2_ of the two different carboxylic groups presented in the structure of cellulose citrate: assuming an average value of 2.5 and 3.5, respectively, their sum agrees with that determined experimentally (5.8–6.7 ± 0.2).

To further investigate the interaction mechanism of the two metals with CC, speciation analysis was performed under the same conditions as the experiment shown in [Fig fig3]. The distribution diagram (Figure S3) indicates that, in the pH range 2–6, both yttrium and neodymium are present in solution as Y­(III) and Nd­(III), which therefore represent the species adsorbed on the surface of cellulose citrate.

Figure 3C,D and Figure S4 show the results of the SEM-EDX analysis performed on randomly selected areas of the samples. In the EDX pattern of the pure cellulose citrate sample, given in Figure S4, the only elements observed were carbon and oxygen.^83^ In CC samples after adsorption, the EDX spectra evidence, in addition, the presence of Y and Nd peaks (Figure S4). In [Fig fig3], it can be observed that the surface of CC is homogeneously covered by the two metal cations.

#### 3.4.2 Effect of Contact Time and Adsorption Kinetics Modeling

The influence of contact time on the adsorption process was assessed with a 6.9 μM solution of the metal at pH 4.5, with the removal efficiency and adsorption capacity being monitored over a period of 400 min. The results are illustrated in [Fig fig4]. Cellulose citrate adsorbs both metals very rapidly in the first 15 min, with *Q*_15 min_ equals to (20 ± 2) μmol Y g^–1^ and (12 ± 3) μmol Nd g^–1^, corresponding to removal efficiency of (58 ± 4) % for yttrium and (40 ± 10) % for neodymium. The initial rapidity of the process can be attributed to the high availability of active sites that can interact with the target metals, also favored by the pH of the solution under investigation. After this time, the rate of adsorption slows due to the progressive decrease in the number of free interacting sites, until the process reaches saturation after 60 min. The kinetics experiments again confirm the slight preference of the material toward Y, with an RE of (76 ± 3) % and *Q_e_* = (26.3 ± 0.4) μmol g^–1^.

**Figure 4:**
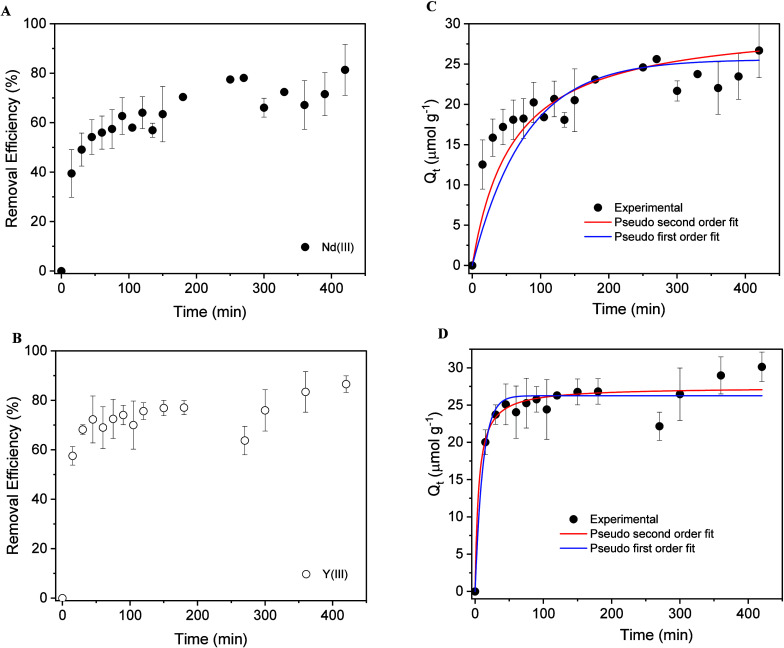
Effect of contact time on removal efficiency (RE%) of Nd­(III) (A) and Y­(III) (B). Adsorption capacity (*Q_t_*) vs contact time for Nd­(III) (C) and Y­(III) (D). The continuous lines are the fitting curves using a pseudo-first and pseudo-second-order model.

For the study of the adsorption mechanism, the collected data were fitted according to two kinetics models: pseudo-first order (PFO, eq 6),^84^ and pseudo-second order (PSO, eq 7),^85,86^ where *k*_1_ (min^–1^) and *k*_2_ (g μmol^–1^ min^–1^) are the pseudo-first-order and the pseudo-second-order rate constants, respectively.

$$Q_{t}=Q_{e}\left(1-e^{-k_{1}t}\right)$$ 6

$$Q_{t}=\frac{Q_{e}^{2}k_{2}t}{1+q_{e}k_{2}t}$$ 7

The fitting parameters are listed in Table 3. Both models describe the adsorption kinetic data well (*R* ^2^ > 0.88), with a better fit to the PFO model for Nd adsorption. The key finding from the analysis is that yttrium adsorbs significantly faster than neodymium. This is evidenced by the kinetic rate constants for Y­(III), which are nearly an order of magnitude higher than those for Nd­(III) in both models, despite their similar equilibrium adsorption capacities (Table 3).

**Table 3:** Fitting Parameters of the Pseudo-First-Order (PFO) and Pseudo-Second-Order (PSO) Models

		parameters	Y	Nd
single metal experiment	PFO	*R* ^2^	0.981	0.921
		*Q*_*e*_ (μmol/g)	26.3 ± 0.3	25.6 ± 0.3
		*k*_1_ × 10^2^ (min^–1^)	9 ± 1	1.3 ± 0.1
		*R* ^2^	0.984	0.884
	PSO	*Q*_*e*_ (μmol/g)	27.4 ± 0.5	30 ± 1
		*k*_2_ × 10^3^ (g μmol^–1^ min^–1^)	7 ± 2	0.6 ± 0.1
competitive experiment Y + Nd	PFO	*R* ^2^	0.987	0.921
		*Q*_*e*_ (μmol/g)	16 ± 1	18 ± 1
		*k*_1_ × 10^2^ (min^–1^)	3.0 ± 0.5	3.6 ± 0.7

#### 3.4.3 Adsorption Isotherms

The influence of the initial adsorbate concentration on the adsorption efficiency was investigated in the range of 6.9–345 μM. The results are shown in Figure 5, where the adsorption capacity (*Q*_*e*_) is plotted as a function of the metal concentration (*C*_*e*_) at equilibrium. Adsorption isotherms show a rapid increase in adsorption capacity as the metal concentration increases from 6.9 to 34.5 μM, particularly for Y. Thereafter, it continues to increase, albeit at a slower rate, until it reaches a plateau, with a *Q*_*e*_ of (153 ± 12) and (141 ± 18) μmol g^–1^ for neodymium and yttrium, respectively. These values indicate that the active sites of CC are more easily saturated by Y. Consequently, a concentration-dependent behavior of cellulose citrate can be observed: at low values, the material interacts more with Y, while, as the amount of the adsorbate increases, the adsorption of Nd becomes more favored. Considering that the carboxylate group may be bonded to REE metal cations in several ways,^22^ the higher adsorption capacity for neodymium compared to yttrium suggests a different binding stoichiometry between the two metal cations, with the possibility for yttrium to more easily form complexes with different coordination modes, reflecting higher CC/M stoichiometric ratios.^22,87^

**Figure 5:**
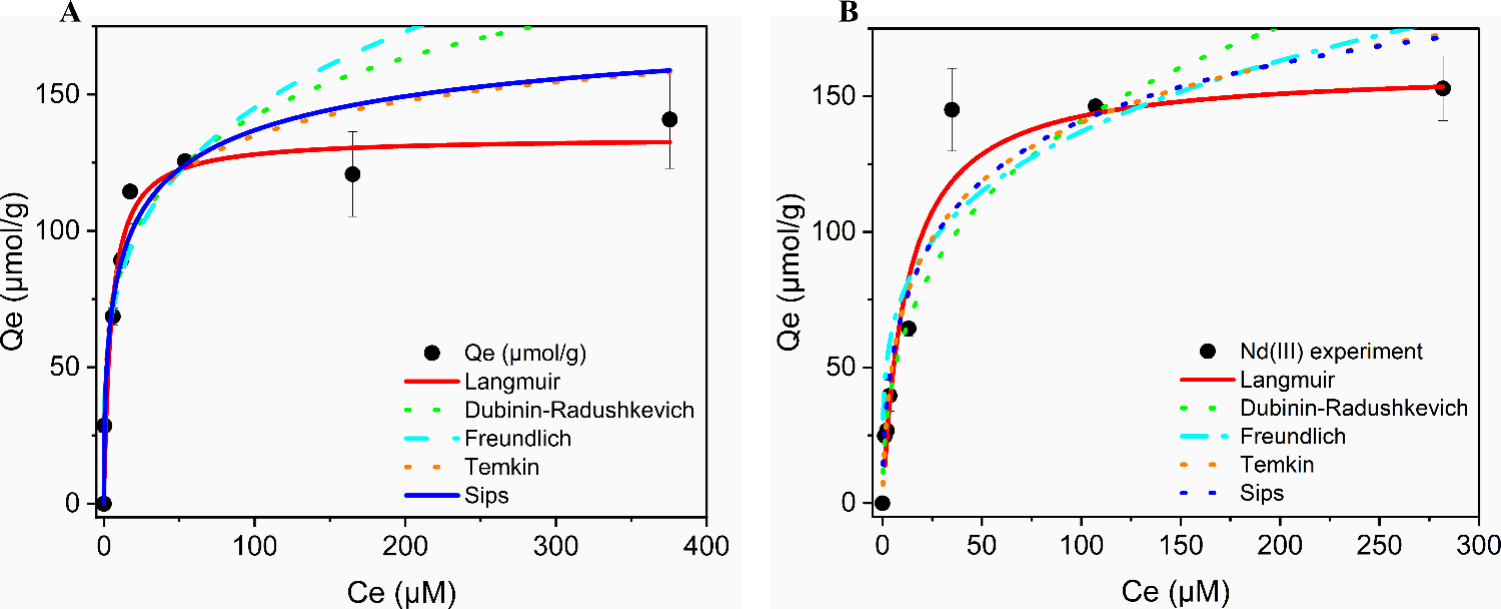
Adsorption isotherms at 298 K of (A) Y­(III) and (B) Nd­(III) by cellulose citrate, fitted using the Langmuir, Freundlich, Dubinin–Radushkevich, Temkin II, and Sips (Langmuir–Freundlich) adsorption isotherm models.

In order to better understand the interaction of the metals with cellulose citrate, the experimental data were fitted according to the following adsorption isotherm models: Langmuir^88^ eq 8, Freundlich^89^ eq 9, Dubinin–Radushkevich^90^ eq 10, and Temkin II^91^ eq 11, defined as

$$Q_{e}=\frac{Q_{\max}K_{L}C_{e}}{1+K_{L}C_{e}}$$ 8

$$Q_{e}=K_{F}C_{e}^{n}$$ 9

$$Q_{e}=Q_{\max}e^{-\beta {\left[RT\ln\left(\frac{C_{s}}{C_{e}}\right)\right]}^{2}}$$ 10

$$Q_{e}=q_{T}\ln\left(1+K_{T}C_{e}\right)$$ 11

where *Q*_*max*_ (μmol g^–1^) is the maximum adsorbent capacity, *K*_*L*_ (μM^–1^) and *K*_*F*_ (μmol g^–1^)­(L μmol^–1^)^*n*^ are the Langmuir and Freundlich adsorption isotherm constants, *n* is a dimensionless number, *q*_*T*_ = *Q*_*max*_/*f*, with *f* being a dimensionless constant given by the sum of the exponential factors of the adsorption and desorption first-order rate equations, and *K*_*T*_ their ratio; β, is a constant related to the mean free energy of adsorption ( $E=1/\sqrt{2\beta }$ ), and *C*_*s*_, the solute solubility. The fitting results are shown in Figure 5, while the fitting parameters obtained are given in Table 4.

**Table 4:** Fitting Parameters of Langmuir, Freundlich, Dubinin–Radushkevich, Temkin II, and Sips (Langmuir–Freundlich) Adsorption Isotherm Models

model	fitting parameter	Y(III)	Nd(IIII)
Langmuir	*Q_max_* (μmol g^–1^)	134 (11)	160 (13)
	*K*_*L*_ (L μmol^–1^)	0.21 (0.08)	0.08 (0.02)
	*R* ^2^	0.926651	0.953341
Freundlich	*K*_*F*_ (μmol g^–1^)(L μmol^–1^)*^n^*	45 (5)	42.9 (0.4)
	*n*	0.25 (0.03)	0.252 (0.002)
	*R* ^2^	0.962462	0.970159
Temkin II	*q*_*T*_ (mol g^–1^)	22 (3)	31.6 (0.2)
	*K*_*T*_ (L μmol^–1^)	6 (3)	0.84 (0.02)
	R^2^	0.993977	0.980867
Dubinin–Radushkevich	*Q*_*max*_ (μmol g^–1^)	387 (40)	683 (73)
	β ×10^9^(mol^2^ kJ^–2^)	1.8 (0.1)	2.8 (0.2)
	*R* ^2^	0.994112	0.991682
Sips (Langmuir–Freundlich)	*Q*_*max*_ (μmol g^–1^)	193 (32)	246.0 (0.8)
	*K*_*L*_ (L μmol^–1^)	0.06 (0.04)	0.0175 (0.0002)
	*n*	0.49 (0.07)	0.526 (0.002)
	*R* ^2^	0.99659	0.999261

While all models provided reasonable fits, their performance varied significantly depending on the concentration range and underlying assumptions. The Langmuir model exhibited the best agreement with the plateau region of the isotherms, where the adsorption capacity approaches saturation. This aligns with its fundamental assumption of monolayer adsorption on homogeneous sites, characterized by a defined maximum capacity (*Q*_*max*_). The fitted *Q*_*max*_ values (Table 4) closely matched the experimental plateaus, supporting their validity at high concentrations. In contrast, the Temkin II and D-R models, despite their high correlation coefficients (Table 4), failed to accurately predict the saturation plateau. The Temkin II model accounts for adsorbate–adsorbate interactions and indirect surface heterogeneity but lacks an explicit saturation term, leading to deviations at high equilibrium concentrations (*Ce*). Similarly, the D-R model, which describes pore-filling mechanisms, overestimated the maximum capacity (Table 4) by assuming infinite micropore availability, a condition not physically realistic in this system. The Freundlich model, while capturing surface heterogeneity through its empirical parameters (*K*_*F*_ and *n*), also struggled to replicate the plateau, as it does not impose an upper limit on adsorption capacity. The divergence between model predictions suggests a dual adsorption mechanism:

1. Low-to-medium *Ce*: Adsorption is dominated by heterogeneous site interactions (Temkin II) or micropore filling (D-R), as evidenced by their superior fits in this region.
2. High *Ce*: The system transitions to homogeneous monolayer coverage, well-described by the Langmuir model.

The binding surface heterogeneity may arise from the presence of different O-binding sites, i.e., carboxylates and hydroxyl groups.

To reconcile the limitations of individual models at different concentration regimes, the Langmuir–Freundlich (Sips) model was evaluated. This hybrid isotherm is expressed as eq 12:

$$Q_{e}=\frac{Q_{\max}{\left(K_{L}C_{e}\right)}^{n}}{1+{\left(K_{L}C_{e}\right)}^{n}}$$ 12

where *n* (heterogeneity index) adjusts the curvature of the isotherm. Unlike the classical Langmuir model, the Sips equation accounts for surface heterogeneity while retaining the concept of monolayer saturation (*Q*_*max*_).^92–94^ The fitting data reported in Table 4 shows that the Sips model outperforms both Langmuir and Freundlich because it captures both the initial curvature (via *n* < 1) and the plateau region (via *Q*_*max*_). In summary, while the Temkin II and D-R models provide excellent fits for the initial adsorption phases, the Langmuir model remains indispensable for describing saturation behavior. The combined modeling strategy using the Langmuir–Freundlich hybrid isotherm equation fully reproduces the adsorption dynamics across all concentrations.

Based on acid–base titration results, the number of binding sites on cellulose citrate was determined to be 1380 μmol/gram (Figure S5), which is significantly higher than the *Q*_*max*_ values for Nd and Y derived from the Sips model (Table 4). A similar finding was reported for *D. dichotoma* stems functionalized with citric acid, which has a maximum adsorption capacity for Ce­(III), almost half that expected considering the total number of carboxylate groups.^95^ However, no explanation for these results was provided. Assuming that each carboxylate group on the CC acts as an active binding site and that metal binding follows a 1:1 stoichiometry, the theoretical-to-experimental *Q*_*max*_ ratios are approximately 1:7 for Y and 1:6 for Nd. These high ratios are clearly unrealistic, suggesting that the actual coordination stoichiometry between the carboxylic groups of cellulose citrate and the metal cations exceeds 1:1. For example, complexes may form in which a single metal ion is coordinated by two carboxylate groups, through either tetradentate or double bidentate modes, potentially involving carboxylates from the same citrate unit or from different ones. Such coordination patterns have been observed for both Nd and Y on rutile (110) surfaces^96^ and likely reflect the necessity to compensate for the high surface charge imparted by trivalent metal cation binding. Additionally, not all carboxylate groups serve as effective binding sites. As indicated by computational results (Section 3.6), complexation via the central COO^–^ group is less favorable than via the lateral one. The key conclusion from this analysis is that Y consumes approximately 1.3 times more binding sites than Nd, indicating a higher coordination stoichiometry for Y, consistent with earlier observations. Consequently, Y­(III) is initially adsorbed to a greater extent due to the high availability of binding sites (low *C*_*e*_) and the greater stability (chelating effect) of the more polydentate complexes, e.g., 1:2. However, it tends to saturate the material more rapidly, as each cation requires a greater number of active sites for coordination.

An examination of the maximum adsorption capacity values reveals that cellulose citrate exhibits intermediate performance compared to the adsorbent materials reported in the literature (Table S4).^25,27,40,42,44–50^ Notably, in studies where the performance of the same adsorbent material was evaluated for both neodymium and yttrium,^25,47–50^ the *Q*_*max*_ values generally fall within the same order of magnitude as those of CC. In many of these works, the primary objective was to develop materials capable of achieving the highest possible selectivity in separating the investigated metals, a challenge that also represents a central focus of the present work. The ratio between the maximum adsorption capacity of Nd and Y may be regarded as an indicator of this selectivity: the greater the deviation of this ratio from unity, the higher the selectivity of the material. Cellulose Citrate is among the materials exhibiting the highest *Q*_*max*_ ratios while maintaining a satisfactory adsorption capacity for each individual metal. Furthermore, our material displays a ratio greater than one (1.27), similar to that of FAU zeolite NaX (1.39),^25^ and is therefore among the few materials demonstrating selectivity toward Nd, which is considered more strategically important than Y in a wide range of technological applications.

At very low concentrations, the adsorption isotherm exhibits a linear trend, which can be used to calculate the distribution coefficient (*K*_*d*_) eq 4, a key parameter for evaluating adsorbent affinity. This linear region reflects Henry’s law-like behavior, where adsorption is directly proportional to the equilibrium concentration (*C*_*e*_). The linear approximation holds when *C*_*e*_ is sufficiently low, ensuring negligible competition for the adsorption sites. Under this condition, the slope of this linear region provides *K*_*d*_ (L g^–1^) defined as eq 13:

$$K_{d}^{Sips}=nQ_{\max}k_{L}^{n}\left(\frac{dQ_{e}}{dC_{e}}=0atC_{e}\to 0\right)$$ 13

The linearity of the isotherms in the low *C*_*e*_ concentration range was checked by the log*Q*_*e*_ *vs*. log*C*_*e*_ data (Figure S6A,B). The slope of both plots for Y and Nd deviates from the Henry’s law regime (slope ≈ 1), indicating the impossibility of extracting *K*_*d*_ from the experimental data. Moreover, the Freundlich exponent obtained by the Sips model (*n* ≈ 0.5) indicates favorable adsorption with moderate heterogeneity, resulting in a concentration-dependent distribution coefficient (eq 13). At infinite dilution, the values of *K*_*d*_ extracted from the Sips model (eq 13) are 71 μmol^0.5^L^0.5^g^–1^ for Y­(III) and 20 μmol^0.5^L^0.5^g^–1^ for Nd­(III), indicating a higher binding affinity of cellulose citrate for yttrium. For practical applications, we also report the *K*_*d*_ values at *C*_*e*_ = 1 μM for Y, 14 μmol^0.5^L^0.5^g^–1^, and for Nd­(III), 11 μmol^0.5^L^0.5^g^–1^ (Figure S6C,D), reflecting the adsorbent’s affinity under trace conditions.

#### 3.4.4 Adsorbent Regeneration and Reusability

The reusability of cellulose citrate was evaluated over two consecutive adsorption–desorption cycles for both yttrium and neodymium, regenerating the material with 0.1 N HCl. Figure 6 illustrates the variation in the adsorption capacity toward Y (Figure 6A) and Nd (Figure 6B) during each reuse cycle as a function of desorption time.

**Figure 6:**
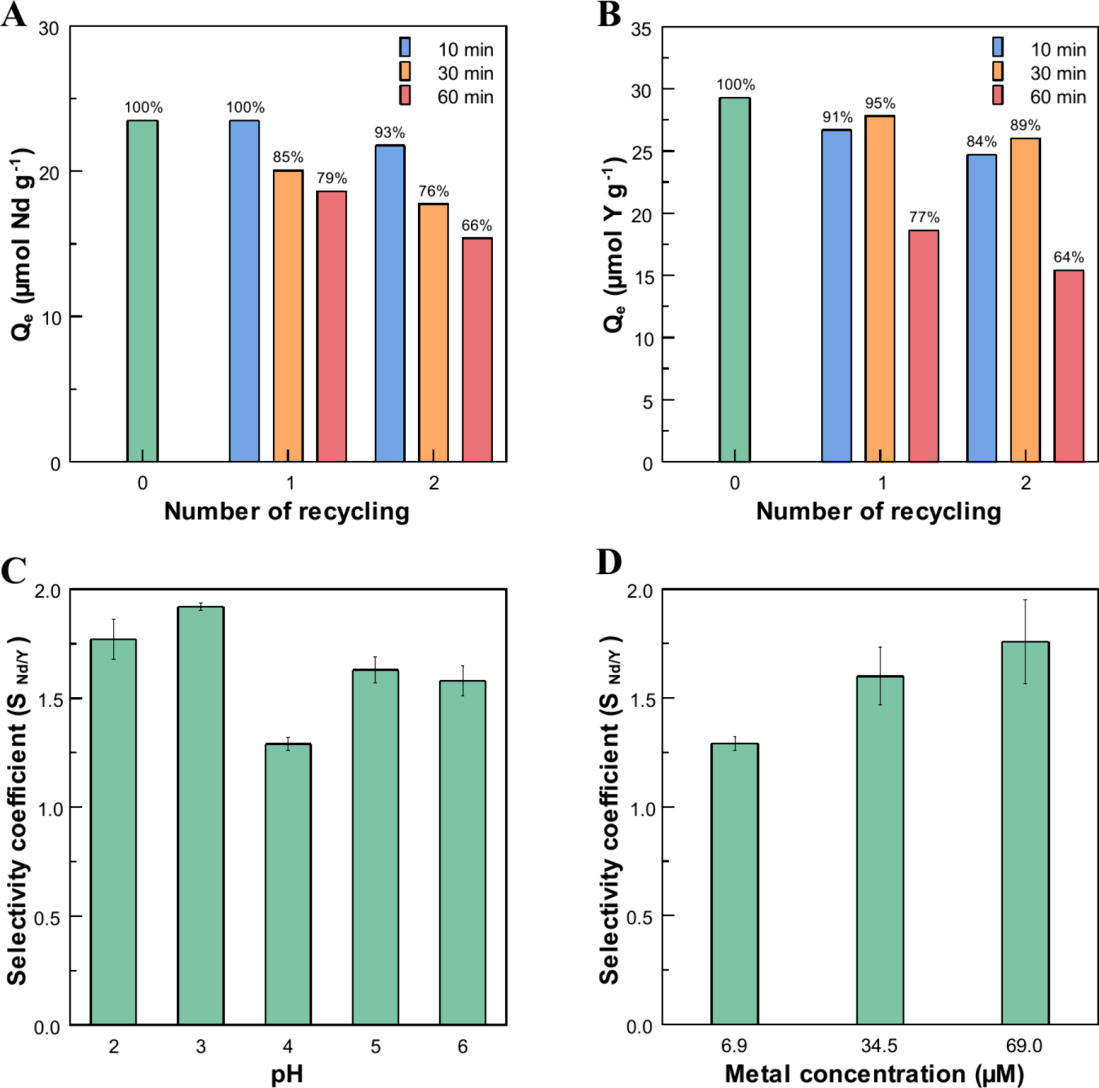
Adsorption capacity of cellulose citrate toward (A) yttrium and (B) neodymium over two consecutive adsorption/desorption cycles as a function of desorption time. Effect of pH (C) and initial metal concentration (D) on the selectivity coefficient (*S*_Nd/Y_).

The results indicate that the adsorption capacity of the material toward yttrium decreases after each reuse cycle, with the extent of variation depending on the desorption time. Specifically, after 10 and 30 min of treatment with the acidic regenerating solution, the reduction in *Q*_*e*_ at the end of the second reuse cycle is minimal and comparable for both times, being 16 and 11%, respectively. Experiments performed with Nd as the adsorbed species show that the optimal desorption time is 10 min, with the adsorption capacity remaining unchanged after the first cycle, followed by a slight decrease of about 7%. Consequently, for short regeneration times, the performance of the cellulose citrate remains comparable to that of its first use. In contrast, long desorption times (e.g., 60 min) lead to a marked decrease in adsorption capacity, which can be attributed to the possible partial hydrolysis of cellulose citrate ester groups, resulting in a reduction of the available active sites.

### 3.5 Selectivity Studies

Due to the pronounced dependence of the distribution coefficient (*K*_*d*_) on metal concentration, the material’s selectivity toward the two metals was assessed by calculating an apparent selectivity coefficient (*S*_Nd/Y_), representative of the actual operating conditions, defined as eq 14:

$$S_{\mathrm{Nd}/Y}=\frac{Q_{e(\mathrm{Nd})}C_{e(Y)}}{C_{e(\mathrm{Nd})}Q_{e(Y)}}$$ 14

#### 3.5.1 Effect of pH, Contact Time, and Initial Metal Concentration

The effect of pH on the selective adsorption of yttrium and neodymium was evaluated in the range of 2–6. The results are illustrated in Figure S7. As seen in experiments with individual metals, adsorption increases with increasing pH. However, when the two REE’s are in competition, cellulose citrate shows a certain degree of selectivity toward Nd, which increases with the acidity of the solution: the selectivity coefficient goes from 1.58 ± 0.07 at pH 6 to 1.92 ± 0.02 and 1.77 ± 0.09 at pHs 3 and 2, respectively (Figure 6C). Looking at the removal efficiency and adsorption capacity values (Figure S7), both metals are adsorbed according to their individual behavior (Figure 3) at pH 5–6. As pH decreases, the fraction of nonionized carboxyl groups increases, making adsorption less favored, as observed in the individual experiments. However, the copresence of the two metals leads to a significant competition between Y and Nd for the available binding sites, resulting in an increase of the selectivity coefficient (Figure 6 Figure 6C). This condition resembles that where the binding sites are almost completely occupied (saturation).

Figure S8 shows the evolution of the removal efficiency and adsorption capacity as a function of contact time during the selectivity experiment. The removal efficiency (Figure S8A) and adsorption capacity at equilibrium (Figure S8B,C) are (56 ± 8) % and (18 ± 3) μmol g^–1^ for Nd, (50 ± 7) % and (16 ± 3) μmol g^–1^ for Y. These values are significantly lower than those obtained during the test with the single metals, indicating a clear competition for the active sites of the cellulose citrate. The overall adsorption capacity is comparable to that observed during the single metal tests. Interestingly, unlike the single metal experiments, the results show that Nd­(III) is adsorbed more rapidly than is Y­(III) under competitive conditions. Notably, the pseudo-first-order rate constant for the adsorption of neodymium is approximately three times higher compared to that observed in the single metal experiment (Table 3). Additionally, only the PFO model accurately fits the experimental kinetic trend (Figure S8B), whereas the PSO model fails to do so (Figure S8C).

The dependence of the initial metal concentration (6.9, 34.5, and 69 μM) on the ability of CC to selectively adsorb yttrium and neodymium was investigated at pH 4.5 under equilibrium conditions. The results are shown in Figure S9. As expected, the removal efficiency decreases with increasing concentration, while the adsorption capacity shows the opposite behavior. The selectivity of cellulose citrate toward Nd increases with increasing concentration and reaches its highest value (*S*_Nd/Y_ = 1.8 ± 0.2) when the solution contains 69 μM of yttrium and neodymium (Figure 6D), in agreement with the isotherm data shown before (Figure 5).

In general, a higher selectivity for Y­(III) might be expected under ideal conditions of infinite dilution (*C*_*e*_ → 0), where the formation of thermodynamically more stable complexes prevails. However, this does not occur in practical applications where both metals must be separated from solutions arising from leachates of i.e., permanent magnets, where significantly higher concentrations are found,^10^ and the greater adsorption capacity of the cellulose citrate toward Nd­(III) begins to prevail.

### 3.6 Computational Outcomes

The cellulose model obtained from MM and DFT optimizations used for the Δ*E*_*ads*_ calculations is shown in Figure 7A. As a result of quantum optimization and the initial MM geometry, with parallel positioned cellulose chains, various hydrogen bonds connecting the chains were found. This represents a satisfactory model for the cellulose citrate.

**Figure 7:**
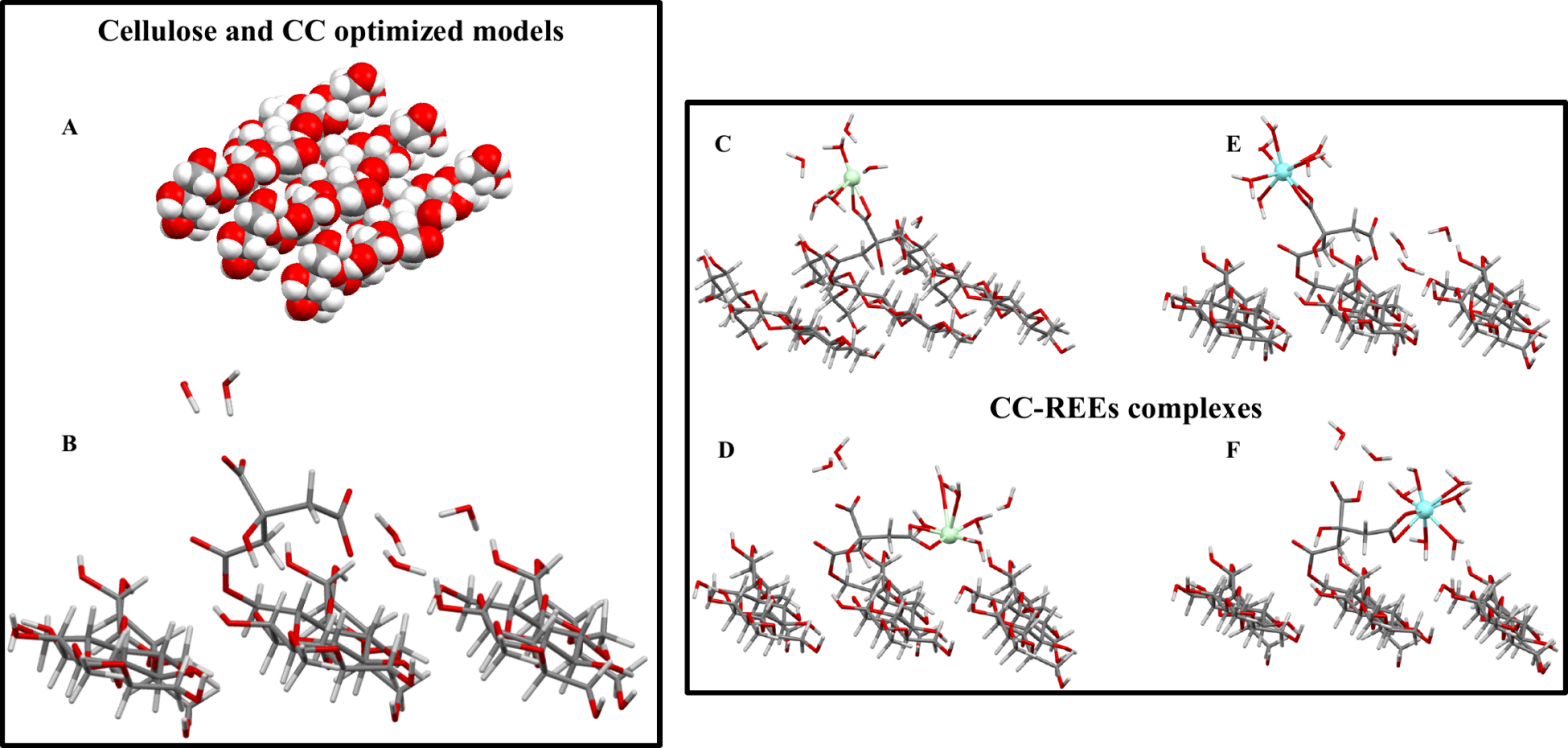
(A) Cellulose model obtained from MM and DFT geometry optimization. Atoms are colored according to the following scheme: carbon (gray), oxygen (red), and hydrogen (white). (B) DFT-optimized structure of the cellulose citrate model used for the ESP calculation. QM/MM optimized structures of the cellulose citrate-metal complexes used in single-point calculations for Δ*E*_*ads*_ calculation at the high DFT level of theory. Nd complex with central (C) and lateral (D) carboxylate ligands; Y complex with central (E) and lateral (F) carboxylate ligands. Nd and Y atoms are colored green and light blue, respectively.

Figure 7 Figure 7B shows the cellulose citrate model, as obtained from the DFT-based optimization and used for the calculation of ESP charges. The citrate was esterified to the cellulosic support using one of the two chemically equivalent carboxyl groups for steric hindrance, as confirmed by the experimental results. Since the calculations referred to solutions at pH > 4, the remaining carboxyl groups were deprotonated. As far as that is concerned, two carboxylates can be identified: the central COO^–^, bound to the C3 atom of citrate, and the lateral one bound to the C4 atom, symmetrically opposite to the esterified carboxyl group. Both the central and lateral carboxylates represent possible metal coordination sites. It is worth noting that in the initial geometry of the CC model (Figure 7 Figure 7B), water molecules between the lateral carboxylate and cellulose were considered in order to take into account the possible interactions between the cellulose chains and the functional group via hydrogen bonds mediated by the water molecules (see H_2_O molecules on the right side of Figure 7 Figure 7B). The lateral COO^–^ actually interacts with the cellulose substrate via hydrogen bonds, as shown in the DFT-optimized geometry; this could make coordination with REEs more difficult on this carboxylate. With the central COO^–^, this does not occur, so it is more available for complexations. It is important to highlight that the interaction between lateral COO^–^ and cellulose affects the atomic point charges of the support. This has been taken into account by using the optimized structures shown in Figure 7 Figure 7B.

Figure 7 Figure 7C–F shows the optimized QM/MM structures of the Nd- and Y-cellulose citrate complexes, considering both the central and lateral carboxylic ligands, respectively. All the structures have been modeled with the metal coordinated by six water molecules. In this case, it was not necessary to include additional water molecules in the coordination of metals with the lateral carboxylate.

In the Nd–central complex, three of the six coordinating water molecules interact at short-range (1.9–2.8 Å), while the remaining molecules interact at long distance (3.1–3.6 Å). In the complex involving the lateral carboxylate, the number of H_2_O coordinating at a short distance increases to five. As for the Y complexes, all six water molecules interact at a short range with the bond distances falling below 2.5 Å.

Figure S10 illustrates the DFT-optimized structures of the [Nd­(H_2_O)_6_]^3+^ (A) and [Y­(H_2_O)_6_]^3+^ (B) aquo-complexes. In both structures, the metals are coordinated with six water molecules arranged on the vertices of an almost ideal octahedron. The corresponding complexation energies (Δ*E*_*M*_(hyd)) for Nd and Y were −749.24 and −532.96 kcal/mol, respectively.

The adsorption energies are reported in Table 5 and can be referred to the adsorption analysis under trace amounts of metal cations. The negative values of the adsorption energies clearly show that Y and Nd are effectively adsorbed on the functionalized cellulose. This means that the energy required to break the M-H_2_O complexes, corresponding to the opposite of the above Δ*E*_*M*_(hyd), is abundantly compensated for from the energy released to form the cellulose citrate-metal complexes. Furthermore, the values in Table 5 shows that the lateral carboxylate is markedly favored over the central one for the formation of the complexes, even though the lateral carboxylate, as previously mentioned, could interact with the cellulose chain through water hydrogen bonds. Finally, the adsorption energy values show that yttrium will be absorbed to a larger extent with respect to Nd at very low equilibrium concentrations of the metals, as prescribed by the distribution coefficients calculated from the Sips isotherms for *C*_*e*_ → 0 (Figure S6). Indeed, the ratio between Δ*E*_*ads*_(Y) and Δ*E*_*ads*_(Nd) is in good agreement with the ratio between the logarithm of the distribution coefficients.

**Table 5:** Energy of Adsorption Referring to Metal’s Adsorption on Central and Lateral Carboxylate Chelating Groups

complex	carboxylate ligands	adsorption energy, Δ*E*_*ads*_ (kcal/mol)	Δ*E*_*ads*_ (Y)*/*Δ*E*_*ads*_(Nd)
cellulose citrate-Y	central	–179.92	1.6
cellulose citrate-Nd		–113.21	
cellulose citrate-Y	lateral	–652.85	1.2
cellulose citrate-Nd		–523.32	

## 4 Conclusions

In this work, cellulose extracted from *Spartium junceum* was functionalized with a citrate group via direct reaction with molten citric acid and subsequently employed as an adsorbent material for the removal of Y and Nd. Preliminary evaluation of the acid constants of citric acid and cellulose citrate by potentiometric titration enabled identification of the carboxylate of the functional group involved in esterification with the cellulose support. The material’s adsorption properties were then evaluated as a function of contact time, pH, and initial metal concentration. CC is a highly efficient material for the adsorption of both metals. The process is pH-dependent, with the most favorable experimental conditions being achieved at values greater than 4. Kinetics experiments showed that equilibrium is reached after just 60 min. Interestingly, a concentration-dependent behavior of cellulose citrate was observed: at low values, the material interacts more with Y, while as the amount of the adsorbate increases, the adsorption of Nd becomes more favored. This finding was attributed to the distinct coordination stoichiometry of the two cations with the carboxylate ligands. In experiments involving both metals in solution, a certain degree of selectivity toward Nd was observed, becoming more pronounced at pH values below 3. All computed adsorption energies give significantly negative values, suggesting that Y and Nd are efficiently adsorbed onto the functionalized cellulose and confirming the experimental findings. In the formation of metal complexes, the lateral citrate carboxylate is markedly favored over the central one, although the former could interact with the cellulose support by water hydrogen bonds.

## Supplementary Material



## Notes

FTIR spectra of cellulosic materials; potentiometric titration data; number of binding site calculation; distribution coefficient determination; pH, contact time, and initial metal concentration effects on removal efficiency and adsorption capacity in selectivity experiments; and DFT-optimized structures of [Nd­(H_2_O)_6_]^3+^ and [Y­(H_2_O)_6_]^3+^(WORD) (PDF)
