# Supplementary material for: Neodymium and Yttrium Adsorption on Citrate-Modified Cellulose: Experimental and Computational Insights
Source: ACS Omega. 2026 Jan 23;11(5):7228–44. doi: 10.1021/acsomega.5c07380 (PMC12903179; doi:10.1021/acsomega.5c07380)
Supplement: Supplementary file 1 [file ao5c07380_si_001.pdf]

**Supporting Information**

# Neodymium and Yttrium adsorption on citrate-modified cellulose: experimental and computational insights

*Alessio C. Perri,[a] Giorgio De Luca,[b*] Nasser AL-Hamdani,[b] Vincenzo Algieri,[a] Emilia Furia,[a] Elpida Piperopoulos,[c] Giuseppina Anna Corrente,[c] Amerigo Beneduci [a*]*

[a] Department of Chemistry and Chemical Technologies, University of Calabria, 87036, Rende, Cosenza, Italy

[b] Institute on Membrane Technology, ITM-CNR, Ponte P. Bucci, cubo 17/c, 87036, Rende, CS, Italy

[c] Engineering Department, University of Messina, C.da di Dio, 98166 Messina, Italy

**Table S1.** XYZ coordinates of the DFT-optimized geometry of the cellulose model

| | | | |
|---|---|---|---|
| O | 8.73908 | 14.26940 | -6.79963 |
| H | 6.92724 | 11.98672 | -3.61224 |
| O | 7.47571 | 9.92137 | -5.11426 |
| H | 8.84809 | 12.07763 | -5.45655 |
| H | 7.98581 | 9.53774 | -5.84786 |
| H | 7.60790 | 14.08627 | -4.40606 |
| C | 6.23447 | 11.85543 | -4.45989 |
| C | 8.03645 | 12.02214 | -6.20068 |
| C | 6.92295 | 11.13957 | -5.61491 |
| O | 5.08265 | 11.16291 | -4.08011 |
| C | 6.89942 | 14.04452 | -5.24584 |
| H | 9.01542 | 11.99647 | -7.88879 |
| O | 5.80913 | 13.15548 | -4.91963 |
| O | 5.65856 | 15.87448 | -4.22513 |
| H | 4.98375 | 15.21844 | -3.96778 |
| O | 8.49806 | 11.34537 | -7.37919 |

| | | | |
|---|---|---|---|
| C | 7.57547 | 13.45833 | -6.48135 |
| C | 6.26908 | 15.41887 | -5.43271 |
| H | 6.17009 | 10.95465 | -6.39320 |
| H | 7.04524 | 16.14309 | -5.69282 |
| H | 6.87248 | 13.45581 | -7.32248 |
| H | 5.54626 | 15.36733 | -6.26481 |
| H | 1.63665 | 10.53311 | 2.32712 |
| H | -0.64864 | 10.60221 | 3.45736 |
| H | 2.87718 | 13.51094 | -1.39077 |
| H | 5.13520 | 12.34747 | -2.34754 |
| H | 6.06779 | 10.47508 | -1.03578 |
| H | -0.33621 | 12.28801 | 1.87362 |
| O | 0.61781 | 8.21860 | 3.20174 |
| H | 2.82183 | 9.55875 | 0.48855 |
| C | 1.18426 | 9.92450 | 1.53136 |
| O | 1.47728 | 11.84197 | 0.02017 |
| C | -1.02493 | 9.91731 | 2.68787 |
| O | 2.14840 | 9.02919 | 0.95923 |
| O | 3.43056 | 10.95886 | -0.80830 |
| C | 2.34757 | 12.76697 | -1.99756 |
| C | 0.09602 | 9.04165 | 2.13632 |
| H | 1.17839 | 14.11231 | -2.81701 |
| C | 4.44840 | 11.67391 | -2.87564 |
| H | 1.37163 | 7.74175 | 2.81305 |
| C | -0.66274 | 11.61115 | 1.06997 |
| H | 5.03272 | 9.08673 | 0.30943 |
| C | 5.38546 | 9.74964 | -1.49720 |
| O | 3.39501 | 13.67720 | -3.92359 |
| O | -2.07002 | 9.11398 | 3.19815 |
| C | 0.53914 | 10.84830 | 0.49916 |
| C | 2.13738 | 11.51324 | -1.15707 |
| C | 4.13007 | 10.46740 | -1.97974 |
| C | 3.16294 | 12.42589 | -3.24330 |
| O | 1.04261 | 13.26184 | -2.36640 |
| O | -1.59774 | 10.65011 | 1.60435 |
| O | 5.06596 | 8.69061 | -0.58504 |
| O | -2.61397 | 12.95865 | 0.44042 |
| H | -0.73625 | 13.22358 | -0.34596 |
| H | 5.88694 | 9.30781 | -2.36280 |
| C | -1.37910 | 12.40210 | -0.02520 |
| H | 4.01135 | 13.47075 | -4.65346 |
| H | -0.33000 | 8.40005 | 1.35530 |
| H | -3.25013 | 12.22561 | 0.36274 |
| H | 0.19321 | 10.23890 | -0.34610 |
| H | 3.48373 | 9.75920 | -2.51774 |
| H | 2.55844 | 11.77226 | -3.88635 |
| H | -1.55748 | 11.74831 | -0.88956 |
| H | 1.57663 | 10.76464 | -1.73808 |
| H | -4.37717 | 10.31361 | -1.36508 |
| H | -5.21108 | 11.56594 | -2.90083 |
| O | -4.05515 | 11.20643 | -1.18334 |
| O | -6.71166 | 12.13763 | -0.88197 |
| C | -4.67092 | 12.11976 | -2.12536 |
| O | -2.86342 | 12.14018 | -3.67337 |
| H | 3.12003 | 12.66515 | -9.59252 |
| C | -5.66796 | 12.97699 | -1.34771 |
| H | -4.79394 | 13.78651 | -4.39447 |
| C | -3.59879 | 12.98410 | -2.78183 |
| H | -2.31027 | 15.14620 | -7.84647 |
| H | -2.06310 | 12.62516 | -3.95111 |
| H | -1.37739 | 13.06809 | -6.60142 |
| H | -3.22602 | 14.12396 | -5.74178 |
| O | -6.25314 | 13.96391 | -2.19847 |
| O | 2.58879 | 13.14347 | -8.93372 |
| H | 1.42117 | 14.07258 | -10.41095 |
| C | -4.25602 | 14.16503 | -3.51472 |
| O | 0.20303 | 14.48259 | -8.16755 |
| C | 2.11080 | 14.31948 | -9.59299 |
| H | -5.16363 | 13.46963 | -0.50951 |
| C | -1.64884 | 15.77283 | -7.22682 |
| H | -0.67241 | 16.83979 | -8.53296 |
| C | -0.69695 | 13.74474 | -6.06334 |
| O | -3.64964 | 16.70528 | -6.22237 |
| C | -2.62872 | 14.82786 | -5.14399 |
| H | -2.95632 | 13.39572 | -1.99375 |
| C | -5.24011 | 14.91300 | -2.61321 |

| | | | |
|---|---|---|---|
| H | -6.54823 | 15.73363 | -4.15691 |
| O | 0.27064 | 11.91682 | -4.75024 |
| O | 3.82878 | 14.38061 | -11.24546 |
| H | 1.00662 | 12.54577 | -6.56240 |
| C | -0.38258 | 14.96858 | -6.93082 |
| O | -1.38789 | 17.01642 | -7.88931 |
| C | -2.38285 | 16.10961 | -5.92981 |
| C | 0.56389 | 12.98304 | -5.66339 |
| O | -3.25697 | 15.12385 | -3.93906 |
| O | -1.34116 | 14.20982 | -4.85386 |
| C | 1.35317 | 15.15339 | -8.56691 |
| C | -5.95303 | 16.08414 | -3.28235 |
| H | -3.45909 | 17.40287 | -6.87254 |
| H | -7.62739 | 16.25669 | -2.31803 |
| C | 3.28472 | 15.13019 | -10.14918 |
| H | 4.37007 | 15.00177 | -11.76698 |
| H | 0.29450 | 12.30824 | -3.85400 |
| H | 2.24009 | 16.44712 | -11.47545 |
| H | -4.71636 | 15.29604 | -1.72467 |
| O | -6.77552 | 16.76365 | -2.37154 |
| O | 0.94371 | 16.40238 | -9.18836 |
| H | 2.00348 | 15.38747 | -7.70835 |
| C | 2.86137 | 16.53271 | -10.57701 |
| H | -5.20473 | 16.80776 | -3.66951 |
| H | 0.33877 | 15.60859 | -6.40627 |
| H | 4.03525 | 15.23063 | -9.34781 |
| H | -1.75935 | 16.77724 | -5.31953 |
| H | 1.29609 | 13.67856 | -5.23136 |
| C | 2.07358 | 17.25455 | -9.49127 |
| O | 4.06203 | 17.28889 | -10.87034 |
| H | 0.95401 | 18.53202 | -10.86523 |
| C | 1.55092 | 18.62346 | -9.92821 |
| H | 2.68681 | 17.38609 | -8.58773 |
| H | -0.12991 | 18.88991 | -8.99790 |
| O | 0.79384 | 19.24628 | -8.92508 |
| H | 2.41717 | 19.27940 | -10.15712 |
| H | 4.90956 | 4.41177 | 7.55576 |
| H | 12.39807 | 6.47799 | -1.46597 |
| H | 4.38388 | 5.41021 | 5.63497 |
| O | 5.49105 | 5.17203 | 7.40087 |
| O | 11.87512 | 6.88504 | -0.75480 |
| C | 4.82345 | 6.01508 | 6.43881 |
| O | 6.85812 | 6.15981 | 5.12658 |
| H | 10.68956 | 8.02358 | -2.05986 |
| O | 2.70837 | 5.96101 | 7.59078 |
| H | 8.32198 | 6.96860 | 2.08998 |
| H | 7.08082 | 5.41777 | 5.71462 |
| O | 10.09135 | 6.21374 | 3.89337 |
| O | 13.09316 | 8.23845 | -2.97028 |
| C | 11.43774 | 8.14518 | -1.26462 |
| H | 10.79325 | 6.88614 | 2.10367 |
| H | 7.17989 | 8.82247 | 0.47011 |
| O | 9.61845 | 8.30879 | 0.28189 |
| C | 5.88929 | 6.93299 | 5.84396 |
| H | 6.39933 | 7.76733 | 2.76070 |
| C | 3.70436 | 6.83787 | 7.06997 |
| H | 4.84467 | 7.33716 | 4.02330 |
| C | 8.89466 | 7.80615 | 2.51413 |
| C | 10.23765 | 7.27004 | 2.98427 |
| H | 13.65612 | 8.86351 | -3.46377 |
| C | 12.62863 | 8.94123 | -1.80674 |
| H | 9.83805 | 6.61894 | 4.76367 |
| C | 5.21205 | 7.90950 | 4.88603 |
| O | 3.08580 | 7.61358 | 6.03924 |
| C | 10.79733 | 8.92024 | -0.12288 |
| C | 9.05166 | 8.91073 | 1.47871 |
| C | 7.71525 | 9.54145 | 1.10324 |
| C | 6.82813 | 8.64363 | 3.26948 |
| O | 8.18677 | 8.36118 | 3.63874 |
| H | 11.57885 | 10.42110 | -2.96395 |
| H | 6.36955 | 7.50171 | 6.65528 |
| H | 4.08536 | 7.49236 | 7.86139 |
| H | 8.54505 | 10.57442 | -0.32435 |
| H | 13.41386 | 8.94597 | -1.03429 |
| C | 12.25894 | 10.39740 | -2.10417 |
| C | 4.00658 | 8.61418 | 5.53803 |

| | | | |
|---|---|---|---|
| H | 10.85415 | 8.08394 | 3.43157 |
| O | 7.89624 | 10.78093 | 0.37662 |
| O | 10.43421 | 10.23785 | -0.59165 |
| C | 6.83577 | 9.84581 | 2.31703 |
| H | 3.20448 | 8.92740 | 3.55419 |
| O | 5.47230 | 10.08808 | 1.88633 |
| O | 6.09861 | 8.95095 | 4.42445 |
| H | 11.51013 | 9.02955 | 0.71134 |
| C | 3.29108 | 9.48516 | 4.49617 |
| H | 9.72192 | 9.68606 | 1.86822 |
| O | 13.47227 | 11.13288 | -2.40331 |
| C | 11.58268 | 11.05205 | -0.90466 |
| H | 1.38465 | 9.27227 | 4.63990 |
| H | 4.34325 | 9.23882 | 6.37894 |
| H | 5.44799 | 10.96957 | 1.47822 |
| H | 7.22125 | 10.71461 | 2.86183 |
| O | 2.01611 | 9.95614 | 4.93231 |
| H | 10.35626 | 12.43174 | -2.00774 |
| H | 3.92558 | 10.34970 | 4.28997 |
| C | 11.04189 | 12.45579 | -1.14388 |
| H | 12.26834 | 11.07382 | -0.04523 |
| H | 9.69620 | 12.32979 | 0.27011 |
| O | 10.39858 | 12.96071 | 0.02778 |
| H | 11.86763 | 13.13349 | -1.37457 |
| C | 13.38870 | 11.79038 | -3.64998 |
| H | 14.32231 | 12.36874 | -3.80466 |
| H | 12.54607 | 12.51488 | -3.67003 |
| H | 13.30938 | 11.05835 | -4.48264 |
| C | 8.58586 | 14.93262 | -8.03610 |
| H | 9.47951 | 15.56681 | -8.20716 |
| H | 7.70135 | 15.60587 | -8.02675 |
| H | 8.52921 | 14.20465 | -8.87425 |
| C | 4.03806 | 17.82826 | -12.17597 |
| H | 4.95757 | 18.43084 | -12.32277 |
| H | 3.17297 | 18.51033 | -12.31945 |
| H | 4.04260 | 17.02445 | -12.94308 |
| C | -7.53769 | 12.83604 | 0.02548 |
| H | -8.29719 | 12.12928 | 0.41752 |
| H | -8.08836 | 13.65889 | -0.47961 |
| H | -6.96295 | 13.20751 | 0.90287 |
| C | -2.98177 | 9.89745 | 3.93934 |
| H | -3.78078 | 9.23030 | 4.32287 |
| H | -3.47696 | 10.66209 | 3.30069 |
| H | -2.49351 | 10.35909 | 4.82669 |
| C | 1.75801 | 6.68687 | 8.34070 |
| H | 1.02267 | 5.96840 | 8.75748 |
| H | 1.19145 | 7.39697 | 7.70065 |
| H | 2.22983 | 7.20253 | 9.20676 |

**Table S2.** XYZ coordinates of the most stable conformer of citric acid methyl ester obtained from the conformational search

| | | | |
|---|---|---|---|
| H | -0.06800 | -2.59095 | 1.68746 |
| C | 0.40385 | -2.10811 | 2.57328 |
| H | 1.19217 | -2.80174 | 2.93711 |
| C | -0.71342 | -1.95980 | 3.65820 |
| O | -1.73501 | -1.15987 | 3.09747 |
| H | -1.35339 | -0.27973 | 2.83745 |
| C | -0.16541 | -1.26156 | 4.90203 |
| O | 0.87874 | -1.77435 | 5.58107 |
| O | -0.65048 | -0.20711 | 5.28360 |
| C | -1.42587 | -3.29852 | 4.02546 |
| H | -2.14797 | -3.08309 | 4.84449 |
| H | -0.65985 | -3.99191 | 4.43893 |
| C | -2.14986 | -4.06832 | 2.95198 |
| O | -1.54085 | -4.56028 | 2.01290 |
| O | -3.47687 | -4.29434 | 3.07124 |
| C | 1.06571 | -0.84592 | 2.07513 |
| O | 0.37545 | 0.03104 | 1.57230 |
| O | 2.41226 | -0.71136 | 2.12179 |
| C | 3.15367 | -0.76159 | 3.28278 |
| H | 3.00331 | -1.74833 | 3.76683 |

| | | | |
|---|---|---|---|
| H | 2.81950 | 0.03413 | 3.98234 |
| H | 4.23353 | -0.63503 | 3.05496 |

**Table S3.** XYZ coordinates of the DFT-optimized geometry of citric acid methyl ester

| | | | |
|---|---|---|---|
| H | 0.07844742 | -3.15814848 | 1.88777907 |
| C | 0.62376114 | -2.44862991 | 2.51991362 |
| H | 1.46698619 | -2.95829957 | 3.00433516 |
| C | 0.34892081 | -2.00463327 | 3.64919843 |
| O | 0.97464071 | -0.76996986 | 3.28630632 |
| H | 0.90584197 | -0.30538352 | 4.15924702 |
| C | 0.39063882 | -1.76179233 | 5.02336027 |
| O | 1.23810589 | -2.60327918 | 5.40364010 |
| O | 0.02443786 | -0.69882448 | 5.62304401 |
| C | 1.40271916 | -3.10988898 | 3.93242622 |
| H | 2.05251641 | -2.72426567 | 4.72720767 |
| H | 0.84723523 | -3.96428873 | 4.34753850 |
| C | 2.31317296 | -3.65889418 | 2.77611357 |
| O | 1.71910759 | -4.13314360 | 1.76365646 |
| O | 3.54988323 | -3.64816366 | 3.00616550 |
| C | 1.12879819 | -1.36934900 | 1.58394425 |
| O | 0.85424101 | -1.31403807 | 0.40147237 |
| O | 2.04375760 | -0.43423571 | 2.01315833 |
| C | 2.35863898 | -0.29194192 | 3.39727627 |
| H | 2.57964197 | -1.24747326 | 3.88025177 |
| H | 1.53330717 | 0.17312032 | 3.94046061 |
| H | 3.24448584 | 0.35478309 | 3.43230447 |

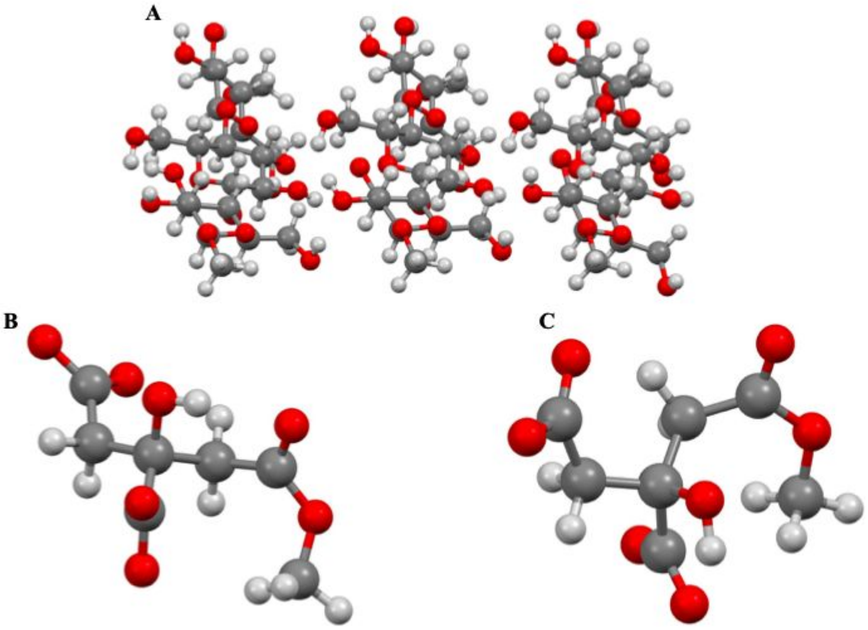


**Figure S1. A** DFT-optimized geometry of the cellulose model, **B** most stable conformer of citric acid methyl ester obtained from conformational search, and **C** its subsequently DFT-optimized geometry

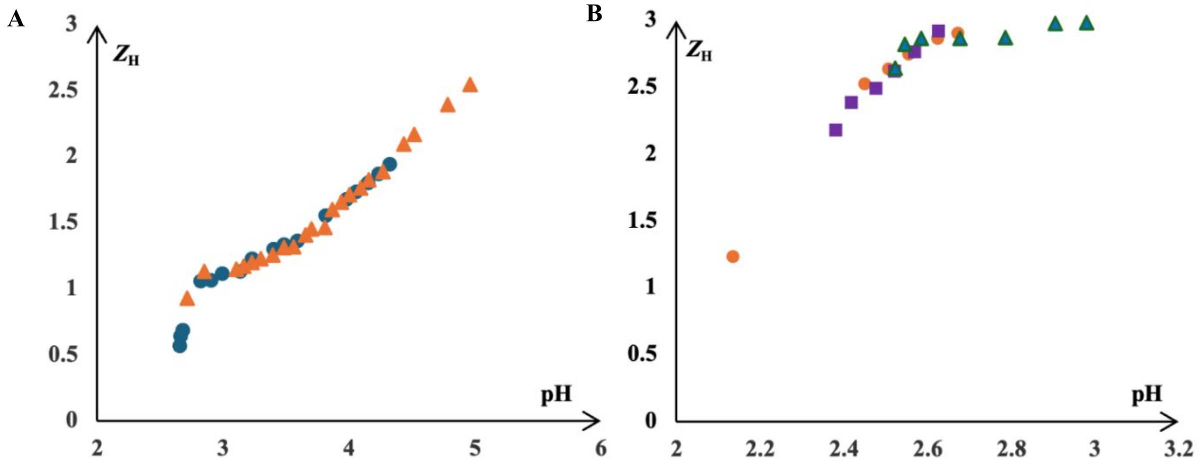


**Figure S2.** ZH as a function of -log $[H^+]$ for **A** citric acid ($H_3L$) and **B** cellulose citrate ($R\text{-}H_2L$) at 25°C and in 0.7 M NaCl. Blue circles and orange triangles in **A** are referred to $C_L$ 1.7 · $10^{-3}$ M and to $C_L$ 0.8 · $10^{-3}$ M, respectively. Purple squares and green triangles in **B** are referred to $C_L$ 1.3 · $10^{-3}$ M and to $C_L$ 0.4 · $10^{-3}$ M, respectively. Orange circles in **B** are back titration points

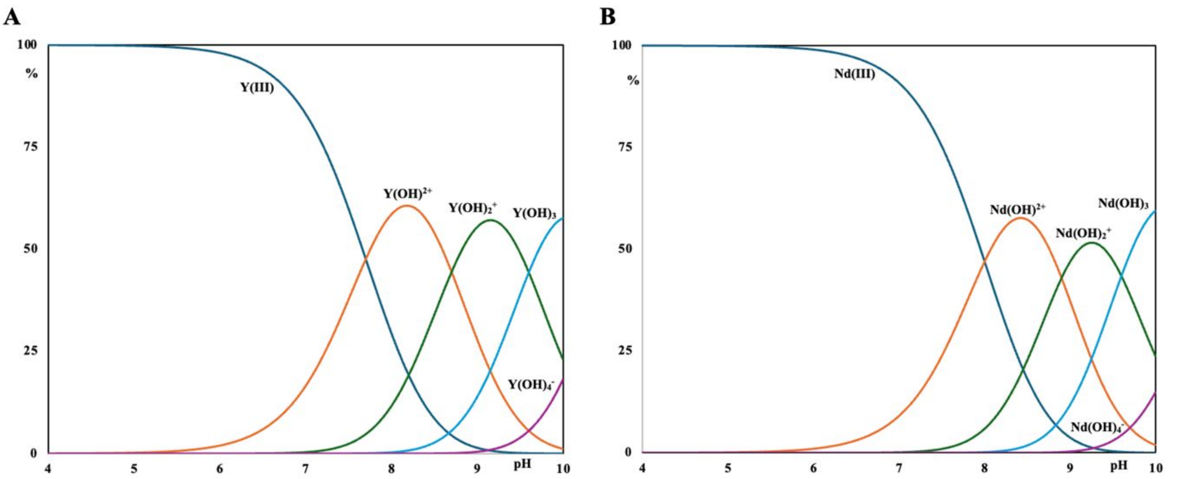


**Figure S3.** Speciation diagram of **A** Y and **B** Nd at 6.9 µM, T = 25°C in ultrapure water. The constants of the predominant hydrolysis products of the Y(III) and Nd(III) cations were taken from the literature(Baes and Mesmer, 1976). In particular, for the Y(III) ion we have considered $Y(OH)^{2+}$ (log $\beta_{11} = -7.7$), $Y(OH)_2^+$ (log $\beta_{12} = -16.4$), $Y(OH)_3$ (log $\beta_{13} = -26.0$) and $Y(OH)_4^-$ (log $\beta_{14} = -36.5$) species, analogously for the Nd(III) ion, $Nd(OH)^{2+}$ (log $\beta_{11} = -8.0$), $Nd(OH)_2^+$ (log $\beta_{12} = -16.9$), $Nd(OH)_3$ (log $\beta_{13} = -26.5$) and $Nd(OH)_4^-$ (log $\beta_{14} = -37.1$)

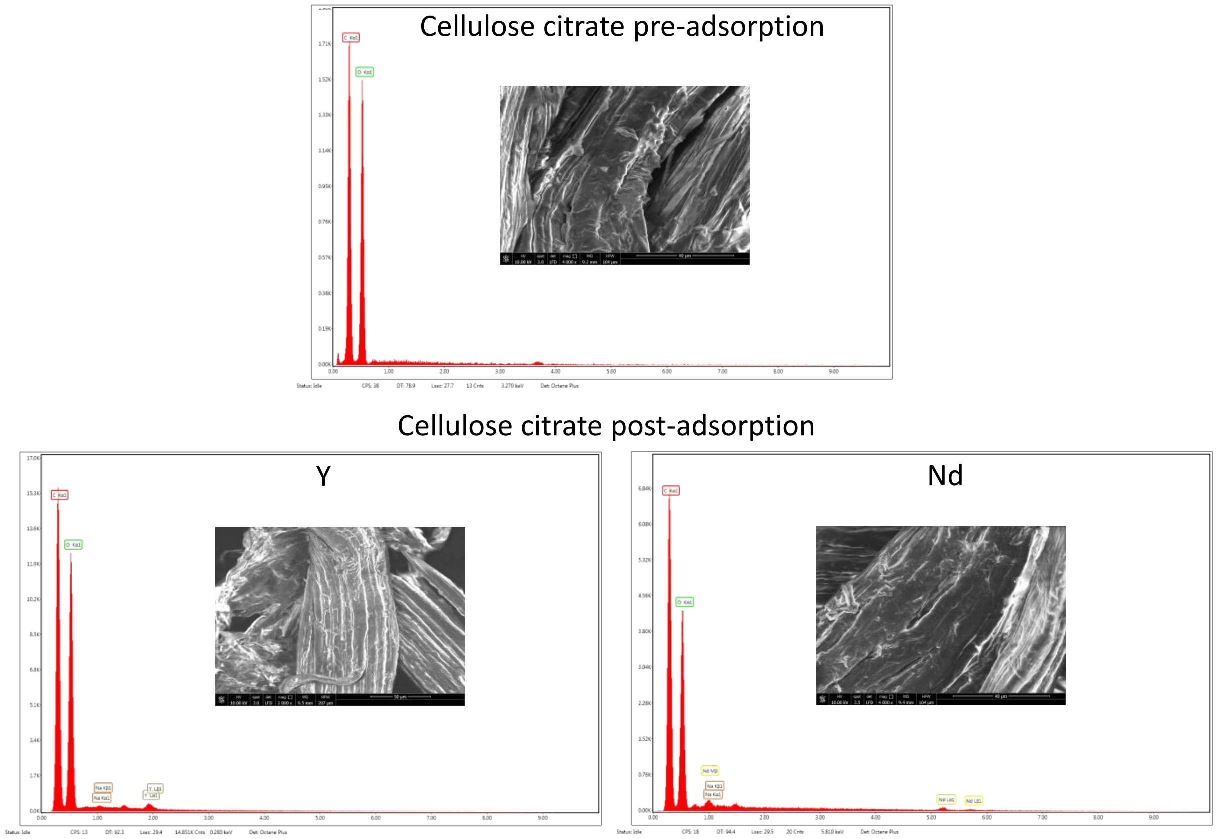


**Figure S4**. EDX spectra of Cellulose citrate, Cellulose citrate after yttrium and neodymium adsorption.

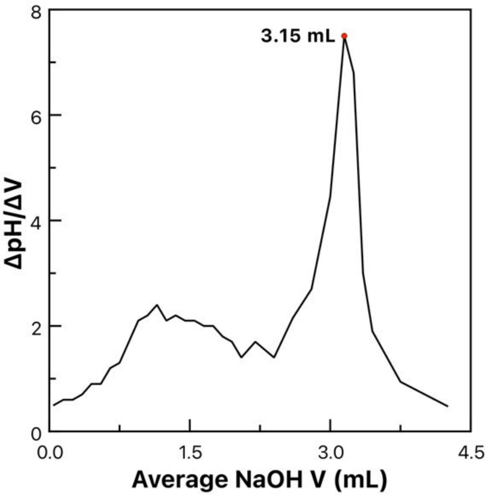


**Figure S5**. First derivative plot used to determine the end point of the acid-base titration (red point)

To reach pH of 2, 3.5 mL of 0.02 M HCl were added. The suspension was then back-titrated with 0.1 M NaOH, reaching the end point at 3.15 mL of titrant (0.340 mmol). After accounting for the unreacted acid, the amount corresponding to the end point was 0.276 mmol. Considering a suspension mass of 0.2 g, the number of binding sites was calculated to be 1380 μmol $g^{-1}$.

**Table S4. Comparison of reported materials for Nd and Y adsorption**

| Adsorbent | REEs | $Q_{max}$ ($\mu$mol/g) | $\frac{Q_{max\,Nd}}{Q_{max\,Y}}$ | Reference |
|---|---|---|---|---|
| **ALG-PGA gel** | Nd | 1650 | | (Wang et al. 2014) |
| **MNHA** | Nd | 2240 | | (Gok 2014) |
| **EDTA-chitosan silica** | Nd | 420 | | (Roosen and Binnemans 2014) |
| **DTPA-chitosan-silica** | Nd | 740 | | (Roosen and Binnemans 2014) |
| **AHIBC** | Y | 1930 | | (Sakr et al. 2021) |
| **Na alginate** | Y | 2040 | | (Khotimchenko et al. 2015) |
| **Ca alginate** | Y | 1110 | | (Khotimchenko et al. 2015) |
| **4As-$TiO_2$** | Y | 1430 | | (Vasylyeva et al. 2021) |
| **Nd/4As-$TiO_2$** | Y | 297 | | (Vasylyeva et al. 2021) |
| **FAU zeolite NaX** | Nd<br>Y | 1320<br>952 | 1.39 | (Guzzinati et al. 2018) |
| **HGF-$GO_{1:1}$** | Nd<br>Y | 338<br>369 | 0.96 | (Xu et al. 2018) |
| **3D $CEG_{1:1}$** | Nd<br>Y | 348<br>362 | 0.96 | (Xu et al. 2019) |
| **GA5MA** | Nd<br>Y | 981<br>1630 | 0.60 | (Iftekhar et al. 2018) |
| **RTCB** | Nd<br>Y | 12<br>20 | 0.60 | (Smith et al. 2016) |
| **Cellulose Citrate** | Nd<br>Y | 246<br>193 | 1.27 | This work |

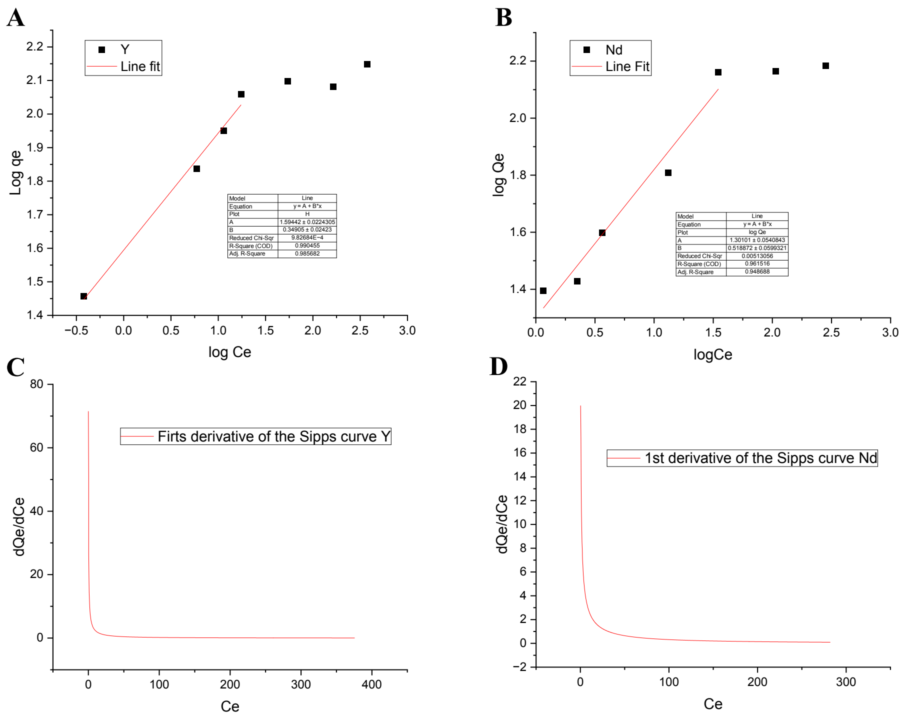


**Figure S6**. Linear fitting of $logQ_e$ $vs.$ $logC_e$ in the low $C_e$ concentration range for **A** Y(III) and **B** Nd(III). First derivate of the Sips curve for **C** Y(III) and **D** Nd(III)

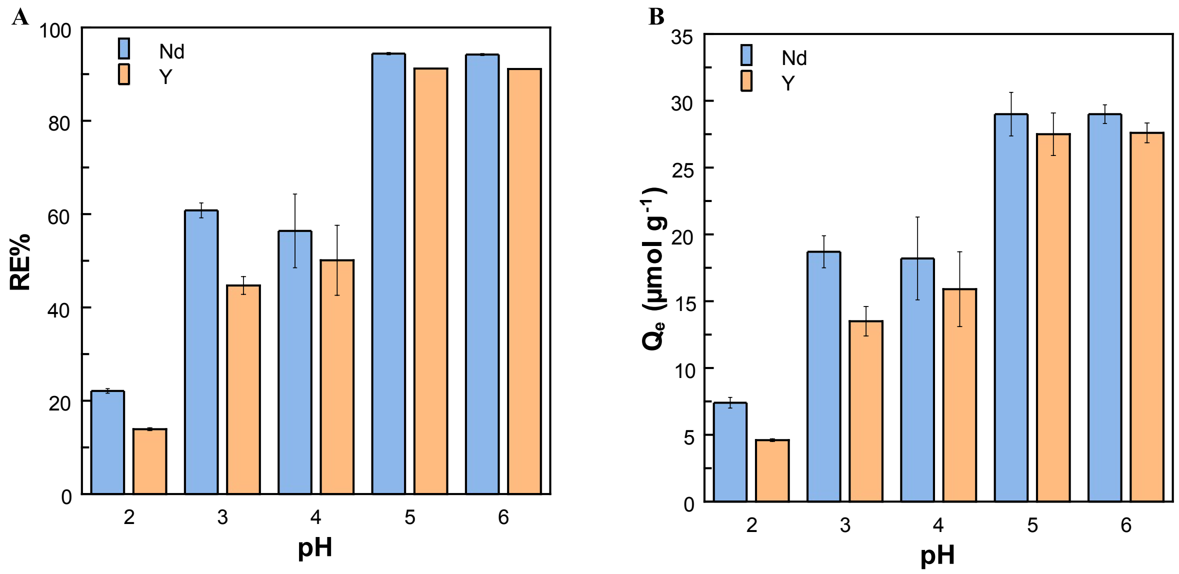


**Figure S7.** Effect of *pH* on **A** removal efficiency (*RE%*), **B** adsorption capacity (*Qe*) during the selectivity experiment

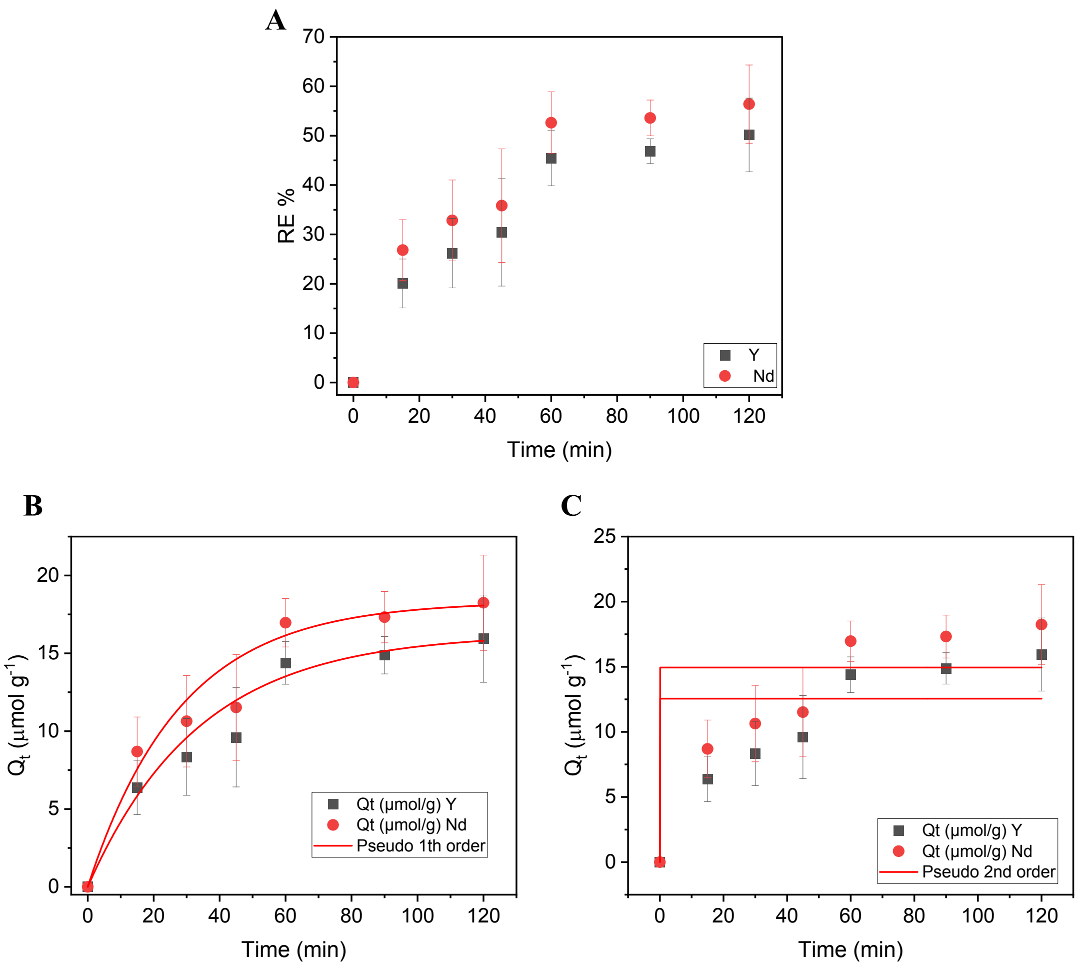


**Figure S8.** Effect of contact time on **A** removal efficiency (*RE%*) during the selectivity experiment. Adsorption kinetics fitted using **B** the pseudo first order and **C** the pseudo second order model

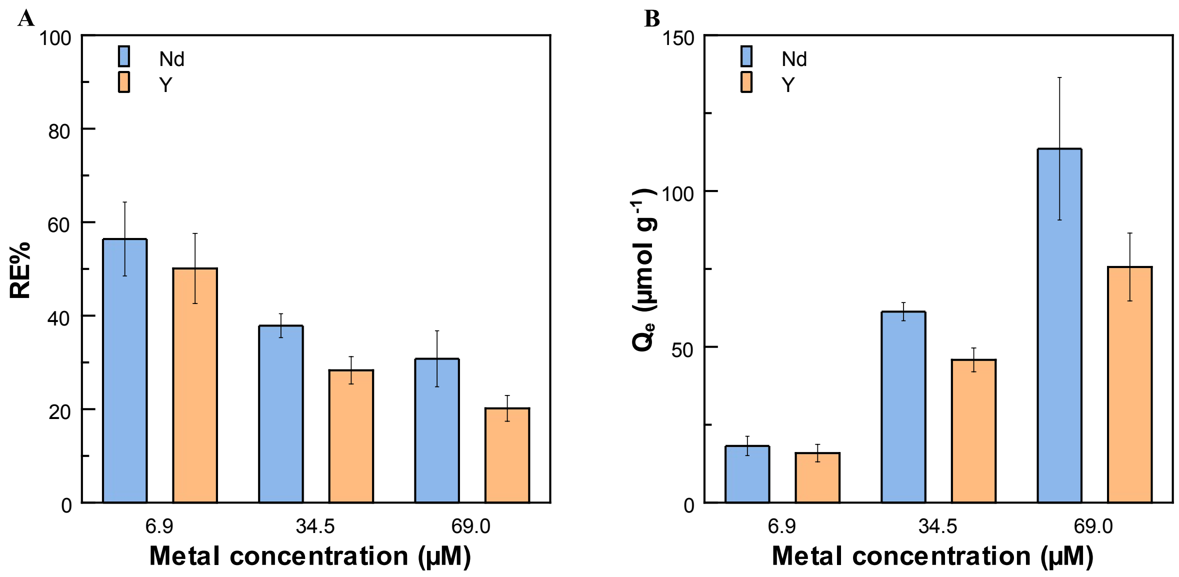


**Figure S9.** Effect of initial metal concentration on **A** removal efficiency (*RE%*) and **B** adsorption capacity (*Qe*) during the selectivity experiment

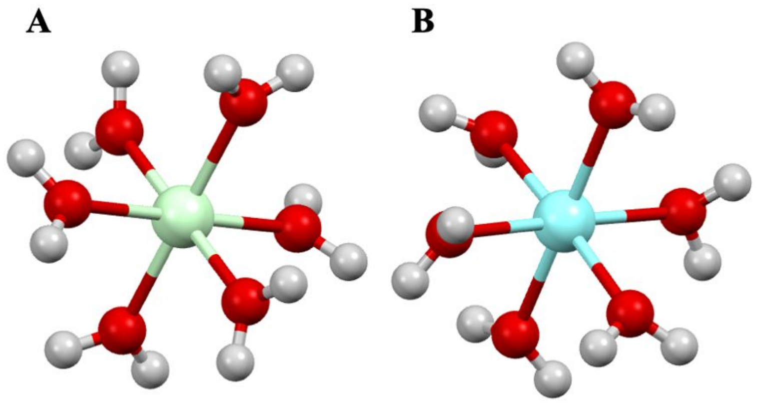


**Figure S10.** DFT optimized structures of hydrated metals $[Nd(H_2O)_6]^{3+}$ **A** and **B** $[Y(H_2O)_6]^{3+}$

## References

Baes CF, Mesmer, RE (1976) The hydrolysis of cations. Wiley Interscience

Gok C (2014) Neodymium and samarium recovery by magnetic nano-hydroxyapatite. J Radioanal Nucl Chem 301:641–651. https://doi.org/10.1007/s10967-014-3193-z

Guzzinati R, Sarti E, Catani M, et al (2018) Formation of Supramolecular Clusters at the Interface of Zeolite X Following the Adsorption of Rare-Earth Cations and Their Impact on the Macroscopic Properties of the Zeolite. ChemPhysChem 19:2208–2217. https://doi.org/10.1002/cphc.201800333

Iftekhar S, Srivastava V, Ramasamy DL, et al (2018) A novel approach for synthesis of exfoliated biopolymeric-LDH hybrid nanocomposites via in-stiu coprecipitation with gum Arabic: Application towards REEs recovery. Chemical Engineering Journal 347:398–406. https://doi.org/10.1016/j.cej.2018.04.126

Khotimchenko M, Kovalev V, Khozhaenko E, Khotimchenko R (2015) Removal of yttrium (III) ions from water solutions by alginate compounds. Int J Environ Sci Technol 12:3107–3116. https://doi.org/10.1007/s13762-014-0737-2

Roosen J, Binnemans K (2014) Adsorption and chromatographic separation of rare earths with EDTA- and DTPA-functionalized chitosan biopolymers. J Mater Chem A 2:1530–1540. https://doi.org/10.1039/c3ta14622g

Sakr AK, Cheira MF, Hassanin MA, et al (2021) Adsorption of Yttrium Ions on 3-Amino-5-Hydroxypyrazole Impregnated Bleaching Clay, a Novel Sorbent Material. Applied Sciences 11:. https://doi.org/10.3390/app112110320

Smith YR, Bhattacharyya D, Willhard T, Misra M (2016) Adsorption of aqueous rare earth elements using carbon black derived from recycled tires. Chemical Engineering Journal 296:102–111. https://doi.org/10.1016/j.cej.2016.03.082

Vasylyeva H, Mironyuk I, Mykytyn I, Savka K (2021) Equilibrium studies of yttrium adsorption from aqueous solutions by titanium dioxide. Applied Radiation and Isotopes 168:. https://doi.org/10.1016/j.apradiso.2020.109473

Wang F, Zhao J, Wei X, et al (2014) Adsorption of rare earths (III) by calcium alginate-poly glutamic acid hybrid gels: Adsorption of rare earths (III) with ALG-PGA. J Chem Technol Biotechnol 89:969–977. https://doi.org/10.1002/jctb.4186

Xu X, Zou J, Teng J, et al (2018) Novel high-gluten flour physically cross-linked graphene oxide composites: Hydrothermal fabrication and adsorption properties for rare earth ions. Ecotoxicology and Environmental Safety 166:1–10. https://doi.org/10.1016/j.ecoenv.2018.09.062

Xu X, Zou J, Zhao X-R, et al (2019) Facile assembly of three-dimensional cylindrical egg white embedded graphene oxide composite with good reusability for aqueous adsorption of rare earth elements. Colloids and Surfaces A: Physicochemical and Engineering Aspects 570:127–140. https://doi.org/10.1016/j.colsurfa.2019.03.022
